# A consensus map for Ug99 stem rust resistance loci in wheat

**DOI:** 10.1007/s00122-014-2326-7

**Published:** 2014-06-06

**Authors:** Long-Xi Yu, Hugues Barbier, Matthew N. Rouse, Sukhwinder Singh, Ravi P. Singh, Sridhar Bhavani, Julio Huerta-Espino, Mark E. Sorrells

**Affiliations:** 1 Department of Plant Breeding and Genetics, Cornell University, Ithaca, NY 14853 USA; 2 International Maize and Wheat Improvement Center (CIMMYT), Apdo. Postal 6-641, 06600 Texcoco, Mexico; 3 Campo Experimental Valle de México INIFAP, Apdo. Postal 10, 56230 Chapingo, Edo de México Mexico; 4 United States Department of Agriculture, Agricultural Research Service, Cereal Disease Laboratory and Department of Plant Pathology, University of Minnesota, St. Paul, Minneapolis, MN 55108 USA; 5 Present Address: United States Department of Agriculture, Agricultural Research Service, Vegetable and Forage Crops Research Unit, 24106 N. Bunn Road, Prosser, WA 99350–9687 USA

## Abstract

*****Key message***:**

**This consensus map of stem rust genes, QTLs, and molecular markers will facilitate the identification of new resistance genes and provide a resource of in** **formation for development of new markers for breeding wheat varieties resistant to Ug99.**

**Abstract:**

The global effort to identify new sources of resistance to wheat stem rust, caused by *Puccinia* *graminis* f. sp. *tritici* race group Ug99 has resulted in numerous studies reporting both qualitative genes and quantitative trait loci. The purpose of our study was to assemble all available information on loci associated with stem rust resistance from 21 recent studies on *Triticum aestivum* L. (bread wheat) and *Triticum turgidum* subsp. *durum* desf. (durum wheat). The software LPmerge was used to construct a stem rust resistance loci consensus wheat map with 1,433 markers incorporating Single Nucleotide Polymorphism, Diversity Arrays Technology, Genotyping-by-Sequencing as well as Simple Sequence Repeat marker information. Most of the markers associated with stem rust resistance have been identified in more than one population. Several loci identified in these populations map to the same regions with known *Sr* genes including *Sr2*, *SrND643*, *Sr25* and *Sr57* (*Lr34*/*Yr18*/*Pm38*), while other significant markers were located in chromosome regions where no *Sr* genes have been previously reported. This consensus map provides a comprehensive source of information on 141 stem rust resistance loci conferring resistance to stem rust Ug99 as well as linked markers for use in marker-assisted selection.

**Electronic supplementary material:**

The online version of this article (doi:10.1007/s00122-014-2326-7) contains supplementary material, which is available to authorized users.

## Introduction

Wheat stem rust caused by the pathogen *Puccinia graminis* Pers. f. sp. *tritici* Eriks. and E. Henn., is one of the most destructive wheat diseases. It can cause up to 90 % yield loss in wheat production but has been effectively under control due to the successful deployment of resistance genes in wheat cultivars since the 1950s (McIntosh et al. 1995). However, the outbreak of a new stem rust race in Uganda named Ug99 (race TTKSK; Pretorius et al. 2000) spread throughout much of Africa, the Middle East and Iran and poses an imminent threat to wheat production worldwide (Singh et al. 2006; Sharma et al. 2013).

To improve the efficiency of wheat breeding for durable resistance to stem rust, it is essential to understand the genetic basis. To-date, 58 stem rust resistance (*Sr*) genes have been numerically designated in wheat as part of the International Wheat Genetics Symposium Gene Catalog (McIntosh et al. 1995, 2011). Several alleles conferring unique race specificities have been identified for many of these genes resulting in a total of 65 numerically designated resistance genes and alleles. Of these genes and alleles, phenotypic data have been published indicating that at least 27 are effective or partially effective to the Ug99 race group: *Sr2* (*Yr30*), *Sr13*, *Sr21*, *Sr22*, *Sr24*, *Sr25*, *Sr26*, *Sr27*, *Sr28*, *Sr32*, *Sr33*, *Sr35*, *Sr36*, *Sr37*, *Sr39*, *Sr40*, *Sr42*, *Sr44, Sr45*, *Sr46*, *Sr47*, *Sr51*, *Sr52*, *Sr53*, *Sr55* (*Lr67/Yr46/Pm46*), *Sr57* (*Lr34*/*Yr18*/*Pm38*), *Sr58* (*Lr46/Yr29/Pm39*) (Faris et al. 2008; Ghazvini et al. 2012; Kolmer et al. 2011; Jin and Singh 2006; Jin et al. 2007; Liu et al. 2011a, b; McIntosh et al. 2012; Rouse et al. 2011; Rouse and Jin 2011; Singh et al. 2013b, c). Several additional resistance genes have been characterized as resistant to Ug99; however, their relationship to numerically designated genes has not been determined: *SrAes7t*, *SrCad*, *SrND643*, *SrTA10171*, *SrTA10187*, *SrTA1662*, *SrTmp*, *SrWeb*, *Sr1RS* ^*Amigo*^ (Hiebert et al. 2010, 2011; Jin and Singh 2006; Jin et al. 2007; Klindworth et al. 2012; Olson et al. 2013). It is possible that such genes with temporary designations are redundant with, or are alleles of, numerically designated genes. Several of the numerically designated *Sr* genes are qualitative and race specific (Jin et al. 2007; Singh et al. 2011). Qualitative genes are phenotyped as present or absent, often at the seedling stage by observing the characteristic low or high infection types displayed by them as described by Jin et al. (2007). One strategy for achieving durable resistance is to pyramid multiple qualitative resistance genes into each wheat variety. A major risk associated with the utilization of qualitative resistance genes is the ability of pathogens to defeat such genes when they are deployed alone in wheat cultivars as has been demonstrated by the original Ug99 defeating *Sr24*, *Sr36*, and resistance in cultivar ‘Matlabas’ resulting in “Boom and Bust” cycles (Jin et al. 2008, 2009; Pretorius et al. 2012).

Utilization of quantitative resistance, often based on multiple minor genes that slow down pathogen infection and colonization in adult plants, referred to as ‘Adult Plant Resistance’ (APR) (Gustafson and Shaner 1982), is another approach for achieving durable resistance. Adult plant resistance to stem rust in wheat is a complex trait conferred by quantitative trait loci (QTL). This type of resistance can be more durable than single gene resistance due to race non-specificity of the resistance genes involved. A total of five numerically designated wheat stem rust resistance genes confer quantitative APR: *Sr2*, *Sr55* (*Lr67/Yr46/Pm46*), *Sr56*, *Sr57* (*Lr34*/*Yr18*/*Pm38*), and *Sr58* (*Lr46/Yr29/Pm39*). In addition, several recent studies have identified numerous QTL associated with wheat stem rust resistance in diverse germplasm (Bansal et al. 2008; Bhavani et al. 2011; Crossa et al. 2007; Kaur et al. 2009; Njau et al. 2013; Rouse et al. 2014; Singh et al. 2013a, b, c; Yu et al. 2011, 2012).

The global effort to identify new sources of resistance to Ug99 has led to the identification of putative new qualitative and quantitative resistance loci reported in numerous studies in the past few years in different populations. As resistance loci are reported, it is important to determine their potential redundancy in order to prioritize those that can be deployed in a breeding program. The purpose of this study was to compile available information on Ug99 resistance loci and their map locations in a single consensus map to facilitate future mapping studies. The map locations from various sources were analyzed along with our recent association mapping projects involving 608 spring and winter wheat breeding lines from CIMMYT and the International Winter Wheat Improvement Program (Yu et al. 2011, 2012). The consensus map contains Diversity Arrays Technology (DArT), Single Nucleotide Polymorphism (SNP), Genotyping-by-Sequencing (GBS) and Simple Sequence Repeat (SSR) markers to facilitate cross-referencing markers and Ug99 resistance loci with other maps.

## Materials and methods

### Literature review and synthesis of stem rust resistance loci

Stem rust resistance loci data were collected from 21 recent studies, for a total of 24 biparental mapping populations, 3 association panels, 2 BC_2_ populations, and two from resistance gene cloning (Bansal et al. 2008; Bhavani et al. 2011; Crossa et al. 2007; Ghazvini et al. 2012; Haile et al. 2012; Hiebert et al. 2010, 2011; Kaur et al. 2009; Letta et al. 2013; Olson et al. 2013; Njau et al. 2013; Periyannan et al. 2013; Rouse et al. 2012, 2014; Saintenac et al. 2013; Singh et al. 2013a, b, c; CIMMYT unpublished, Yu et al. 2011, 2012) (Table 1). The details of the reports, including the chromosome positions of stem rust resistance loci, their LOD (log (Odds)) scores and *p* values, parents used to develop the population as well as stem rust pathotypes for disease reaction are presented in Table 1. Three of the studies did not determine stem rust resistance loci by screening with Ug99 (Bansal et al. 2008; Crossa et al. 2007; Kaur et al. 2009). Though included these loci in our study, their effectiveness against Ug99 is unknown and are presented because of their relevance to Ug99 studies.

**Table 1:** Stem rust resistance loci identified in 21 studies including 24 mapping populations, three association panels, and two BC2 populations

		Sr locus name	Phenotypic variation (R^2)	Chromosome	Start		End		Resistance carrier	Population	References
					Marker	Position cM	Marker	Position cM			
Biparental population		QSr.Sun-5BL	12 %	5BL*	ksuHb9	34.2	glk0354	44.8	Arina	Arina/Forno	Bansal et al. (2008)
		QSr.Sun-7DS	26 %	7DS*	cdo0475b	90.2	gwm1002	94.8	Arina		
	Across all data set	QSr.Sun-3BS	9–15 %	3BS	wPt-8093	31.4	gwm566	61	HD2009	HD2009/WL711	Kaur et al. (2009)
		QSr.Sun-5DL	20–44 %	5DL	gwm182	60.2	SrHD	85.3	HD2009		
		QSr.Sun-7A	7–13 %	7A*	wPt-3992	136.1	gwm260	157.3	HD2009		
	Envir. specific	QSr.Sun-2B	8–13 %	2B	wPt-4892	99	wPt-4892	104.4	HD2009		
		QSr.Sun-5B	11–15 %	5BS*	wPt-6136	6	wPt-5346	34.1	HD2009		
		QSr.Sun-4B	9 %	4B	wPt-5559	6	wPt-8756	8.4	WL711		
		QSr.Sun-lD	12 %	ID*	wPt-4687	4.9	–	–	HD2009		
		*Sr30*	–	5DL*	CFD12	9	gwm292	16	Webster	RL6071/Webster	Hiebert et al. (2010)
		*Sr*Web	–	2BL	gwm47	1.4	wmc332	12.4	Webster		
		–	–	1A	wPt-0128	–	wPt-734078	–	Kingbird	PBW343/Kingbird	Bhavani et al. (2011)
		–	–	3BS	wPt-3921	–	wPt-2757	–	Kingbird		
		–	51.2	5BL	wPt-2607	–	wPt-1733	–	Kingbird		
		–	–	7A	wPt-8670	–	wPt-744574	–	Kingbird		
		–	–	7DS	wPt-1859	–	wPt-731810	–	Kingbird		
		–	6	2D*	barc095	–	–	–	Kiritaki	PBW343/Kiritaki	
		–	25	3BS*	SW58		–	–	Kiritaki		
		–	8	5BL*	barc109		–	–	Kiritaki		
		–	12	7D	Lr34-linked	–	–	–	Kiritaki		
		–	–	2B*	wPt-7829	–	wPt-2266	–	Juchi	PBW343/Juchi	
		–	–	3BS	wPt-8056	–	wPt-800213	–	Juchi		
		–	42.4	4A	wPt-5124	–	wPt-6390	–	Juchi		
		–	–	5BL	wPt-0750	–	wPt-5896	–	Juchi		
		–	–	6B	wPt-5480	–	wPt-9532	–	Juchi		
		–	6.8	2B	–	–	wmc257	–	Huirivis#1	PBW343/Huirivis#1	
		–	16	3BS*	–	–	SW3648	–	Huirivis#1		
		–	23	5BL*	wms371	–	NW2012ND	–	Huirivis#1		
		–	6.9	7B*	–	–	NW3109ND	–	Huirivis#1		
		–	–	2B	wPt-744022	–	wPt-1964	–	Muu	PBW343/Muu	
		–	46	3BS	wPt-666139	–	wPt-3921	–	Muu		
		–	–	5BL	wPt-6014	–	wPt-3661	–	Muu		
		–	–	1BL	wPt-1560	–	wPt-7486	–	Pavon76	Avocet/Pavon76	
		–	68.9	3BS	wPt-8093	–	wPt-7212	–	Pavon76		
		–	–	5A	wPt-6048	–	wPt-4249	–	Pavon76		
		–	–	6B	wPt-1541	–	wPt-0171	–	Pavon76		
		*Sr28*	–	2BL	wcm332	0	wPt-7004	7.6	SD1691	SD1691/LMPG-6	Rouse et al. (2012)
		*Sr42*	–	6BS*	barc183	5.5	FSD_RSA	6	Norin 40	Norin 40/LMPG-6	Ghazvini et al. (2012)
		–	15.1	1BL*	wPt-8894	–	wPt-6015	–	–	PBW343/Kenya Nyangumi	
		–	22.4	2BL	wPt-2274	118.2	wPt-2135	118.2	–		
		–	8.8	3BS	wPt-800213	23.3	wPt-742648	26.7	–		
		–	33.7	5BS*	wPt-8604	25.6	wPt-7542	–	–		
		–	56.9	6BS	wPt-4283	5	wPt-7207	7	–		
		–	6.6	7AS	wPt-742244	2.9	wPt-672171	5.6	–		
		–	10	7BL	wPt-0138	120.6	wPt-4258	–	–		
		–	24.3	1B*	wPt-0320	–	wPt-1911	–	–	PBW343/Cross Bill	
		–	14.8	3BL*	wPt-8105	–	wPt-10003	–	–		
		–	23.5	3BL	wPt-6047	–	wPt-9433	–	–		
		–	55.3	3BS*	wPt-3609L3R3	–	wPt-3609L2R2	–	–		
		–	8.6	6BS	wPt-5461	–	barc79	–	–		
		–	19.4	3BS	wPt-6043	14.2	wPt-742337	15.6	–	PBW343/Diniza	
		–	50.3	3BS*	wPt-2220	67.3	wPt-4364	68.6	–		CIMMYT unpublished
		–	7.8	7AL	wPt-741686	150.9	wPt-7763	168.5	–		
		–	20.9	1AL*	wPt-732616	133.1	wPt-4065	113.4	–	PBW343/Kenya Swara	
		–	22.4	3BS*	wPT-800213	17.1	wPt-3921	26.7	–		
		–	31.2	6AS	wPt-669498	0	wPt-6520	2.5	–		
		–	5.4	7AL	wPt-6495	150.9	wPt-741686	154.8	–		
		–	4.2	7DS*	wPt-743395	–	wPt-742859	–	–	PBW343/Kenya Kudu	
		–	12	1AS	wPt-0512	4.6	wPt-1770	–	–		
		–	17	2BL	wcm474	54.4	wPt-2600	70.4	–		
		–	15	3AL	wPt-9154	73.4	cfd2	–	–		
		–	30	3BS	gwm533	–	wPt-3921	17.1	–		
		–	12	5BL	wPt-0750	102.7	wPt-3114	117.6	–		
		–	10	7BL	wPt-8007	140.8	wPt-5460	145.2	–		
		–	23.8	1BL	wPt-1560	8.6	wPt-7486	–	Pavon76	Avocet/Pavon76	Njau et al. (2013)
		–	6	IDS	wPt-7140	57.1	wPt-5320	60.7	Pavon76		
		–	7	2AL	wPt-7024	71	wPt-7011	76	Pavon76		
		–	5.5	2BL*	wPt-4125	82.7	wPt-1505	102.4	Pavon76		
		–	51.8	3BS	wPt-1081	17.1	wPt-3781	24.9	Pavon76		
		–	18.1	7BL	wPt-4319	143	wPt-3190	–	Pavon76		
		*QSr.cdl*-*1AL*	–	1AL	wPt-0827	–	wPt6869	–	Thatcher	Thatcher/McNeal	Rouse et al. (2014)
		*QSr.cdl*-3BS (*Sr12*)	–	3BS	gpw1120	–	wPt4082	–	Thatcher		
		*QSr.cdl* -*2BS*	–	2BS	wPt-2106	–	cfd238	–	Thatcher		
		*Sr57 (Lr34)*	–	7DS*	csffr6	–	–	–	McNeal		
		*QSr.ipk*-*1A*	3.7	1AL	Xbarcl48	121.6	Xbarc119	137.7	Sebatel	Kristal/Sebatel (durum)	Haile et al. (2012)
		*QSr.ipk*-*2A*	3.9	2AS*	Xgwm448	66	Xgwm1198	79.5	Sebatel		
		*QSr.ipk*-*3B*	8.6	3BS*	Xgwm779	0	Xgwm389	9.6	Sebatel		
		*QSr.ipk*-*4B*	3.8	4BL*	Xgwm1167	24.6	Xgwm1278	83.1	Sebatel		
		*QSr.ipk-5B*	3.7	5BL	Xgwm408	56	Xbarc142	83.1	Sebatel		
		*QSr.ipk*-*6A*	6.5	6AL*	Xgwm494	104.7	Xgwm11150	124.7	Sebatel		
		*QSr.ipk*-*7A.1*	3.9	7A*	Xgwm974	106.3	Xgwm631	120.8	Sebatel		
		*QSr.ipk*-*7A.2*	3.4	7AL	Xbarc121	144.2	Xgwm984	154.4	Sebatel		
		*QSr.ipk*-*7B*	7.3	7 BL*	Xgwm146	127.7	Xgwm344	152.6	Sebatel		
		–	14.5	4B*	wPt-0872	–	–	–	–	Sachem/Strongfield (durum)	Singh et al. (2013a)
		*QSr.spa*-*4B.1*	2.4-6.1 %	4B*	wPt-744434	62.3	Xwmc-617	89.4	Carberry	AC Cadillac/Carberry	Singh et al. (2013b)
		*QSr.spa*-*6D*	3.9-48.8 %	6D*	wPt-664770	0	wPt-1695	9.7	AC Cadillac		
Association mapping			12	1BS	wPt-1560	8.6	wPt-5678	33.7	–	LD-SRRSN (Spring)	Yu et al. (2011)
			7	2BL	wPt-7200	82	wPt-8460	101	–		
			8	3BS	wPt-6945	57	wPt-1940	68	–		
			7	3BS	csSr2	0	wPt-8446	11	–		
			6	4AL	wPt-5857	68	wPt-5749	72	–		
			5	5BS	wPt-1149	31	wPt-5346	33	–		
			5	6AL	wmc417	88	gwm617	97	–		
			5	6AS	wPt-6520	2	wPt-4016	4	–		
			14	6BS	wPt-3774	0	wPt-1922	4	–		
			11	6BS	wPt-5333	31	wPt-5037	57	–		
			12	7BL	wPt-5343	126.1	wPt-7351	126.8	–		
			14	7DL	wPt-1859	116	wPt-7351	126.8	–		
			15	7DL	wPt-7763	160	wPt-664017	175	–		
			8	7DS	wPt-2565	1.4	wPt-665260	5	–		
			6	1AS	wPt-730213	0.2	wPt-730213	0.2	–	LD-SRRSN (Winter)	
			7	2BS	wPt-4916	2	wPt-1964	25	–		
			8	3BS	csSr2	0	wPt-8446	11.4	–		
			5	4AL	wPt-3349	84.5	wPt-7807	87.8	–		
			5	5BS	wPt-1302	1	wPt-3873	27	–		
			5	6BL	wPt-6116	107	wPt-6116	107	–		
			6	6BS	wPt-4930	9	wPt-1241	24	–		
			5	6BS	wPt-4648	51	wPt-4648	51	–		
			6	7BS	wPt-0138	18.9	wPt-0138	18.9	–		
			10	7DS	csLV34	50.5	csLV34	50.5	–		
		Sr31	–	1BS	wPt-8949	11.4	wPt-8616	32.3	–	ESWYT	Crossa et al. (2007)
		Sr19,23,36,40	–	2B	wPt-0100	17.7	–	–	–		
		Sr19,23,36,40	–	2B	wPt-9402	57.8	–	–	–		
		Sr2,12	–	3B*	wPt-0365	44	wPt-8983	44	–		
		Sr2,12	–	3B*	wPt-6802	56.9	–	–	–		
		–	–	4AS	wPt-2788	58	–	–	–		
		–	–	4AL	wPt-2084	161	wPt-7807	161.7	–		
		–	–	4BS	wPt-8650	7.8	–	–	–		
		–	–	5BL	wPt-4996	82.8	–	–	–		
		Sr13, 26	–	6AL	wPt-7623	38	–	–	–		
		–	–	6BS	wPt-7662	36.8	wPt-7777	41.9	–		
		–	–	6BS	wPt-3733	87.5	–	–	–		
		Sr11	–	6BL	wPt-0171	171.4	wPt-1541	181	–		
		Sr17	–	7B	wPt-1149	64.7	–	–	–		
		Sr17	–	7B	wPt-5343	99	–	–	–		
		Sr17	–	7B	wPt-0600	114.3	–	–	–		
		Sr44	–	7D	wPt-2565	1.3	–	–	–		
		–	4.6	1BS*	barc8	32	–	–	–	AM durum panel	Letta et al. (2013)
		–	3.9	2AS*	gwm1045	87.7	–	–	–		
		–	4.1	2BL*	wmc356	220	–	–	–		
		–	3.3	3AS	wPt-7992	8	–	–	–		
		–	4	3AL*	wmc388	85.6	–	–	–		
		–	4.1	5AL*	gwm126	93.3	–	–	–		
		–	4.4	5AL	gwm291	111.7	–	–	–		
		–	3.5	6AL	gwm427	139.5	–	–	–		
		–	7.1	6AL*	CD926040	144	–	–	–		
		–	4.5	6AL*	barc104	155.3	–	–	–		
		–	5.2	7AS*	wPt-2799	38.2	–	–	–		
		–	1.5	7AS*	wPt-7785	94.8	–	–	–		
Direct back crossing		SrTA10187	–	6DS*	Xcfd49	5.4	Xbarc173	20.9	Aa. tauschii- TA10187	BC2-TA10187/KS05HW14	Olson et al. (2013)
		SrTA10171	–	7DS*	–	–	Xwmc827	0.9	Aa. tauschii- TA10171	BC2-TA10171/KS05HW14	

* indicates QTL that are not displayed on the map

### Construction of a consensus map

A consensus genetic map was constructed using the Wheat Interpolated Maps v4 (Diversity Arrays Technology Pty. Ltd., personal communication) as a reference map.

(http://www.triticarte.com.au/). DArT, SNP, GBS and SSR markers from the Wheat KASPar SNP database (http://www.cerealsdb.uk.net/CerealsDB/SNPS/), the 2004 Wheat SSR Consensus Map (Somers et al. 2004), and the Thatcher/McNeal map (Sherman et al. 2013) with concatenated DArT and SSR markers were integrated to the reference map using the ad hoc R package “LPmerge” (Endelman and Plomion 2011) and BioMercator V3.0 (http://moulon.Inra.fr). LPmerge is an optimized “synthetic” (Wenzl et al. 2006) or “composite” (Hudson et al. 2012) approach to built a map across multiple populations. As opposed to minimizing an objective function based on the observed recombination frequencies between markers (JoinMap; Van Ooijen 2006) or (MultiPoint; Ronin et al. 2012), this R package based its algorithm directly on the component linkage maps instead of the recombination frequencies. Other software uses the same approach, but LPmerge implements an additional algorithm for resolving ordinal conflicts found when the marker order was not consistent between the different linkage maps. Resistance loci or QTL associated with stem rust resistance identified from 21 studies and one personal communication (Table 1) were projected onto the consensus map based on the position of the markers linked to the loci when the precision of the constructed map allowed it (Fig. 2). A star following the chromosome number on Table 1 tags the QTL not present on Fig. 2.

**Fig. 1:**
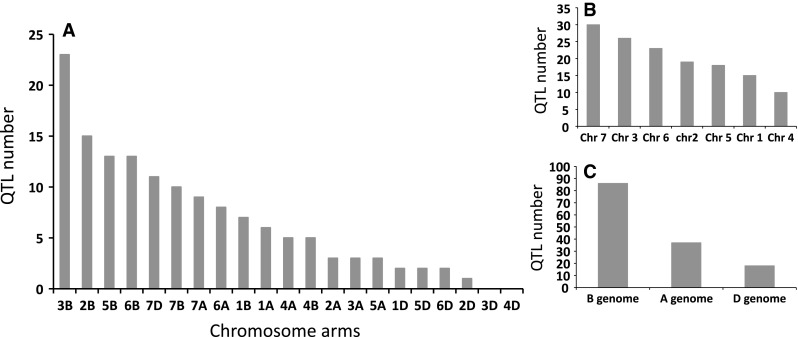
**a** Distribution of QTL associated with stem rust resistance by chromosome arm based on 21 studies. **b** Distribution of QTL associated with stem rust resistance by chromosome based on 21 studies. **c** Distribution of QTL associated with stem rust resistance by genome based on 21 studies

## Results

### Overview of loci for stem rust resistance

The constructed consensus map contains a total of 1,433 markers distributed among the 21 chromosomes. Fourteen percent of these markers are SNP, 16.7 % are GBS, 39.4 % are DArT and 29.9 % are SSR. With a global average distance of 2.7 cM between markers, the maximum average distance between markers is observed on chromosome 5D (16 markers) with 7.6 cM and the minimum average distance between markers is observed on chromosome 3B (158 markers) with 1.9 cM. An expected negative correlation is observed between the marker number per chromosome and average distance in centimorgan between markers (*r* = 0.719). The average chromosome size was 150 cM with a maximum of 203.3 cM for chromosome 7A and a minimum of 83.4 cM. Resistance gene and QTL maps and gene cloning information from 21 studies and one personal communication (Table 1) involving 24 mapping populations, three association panels, and two BC_2_ populations were concatenated to construct a map for stem rust resistance loci (Fig. 1). The map consists of 141 stem rust resistance loci distributed across the genome, many of which were redundant loci detected in at least two studies. A total of 37, 86 and 18 resistance loci were located in the A, B, and D genomes, respectively (Fig. 1). Several hotspots of resistance loci were observed across the genome. Nineteen were located on chromosome arm 3BS, while 6BS, 5BL and 2BL had nine, nine, and seven QTL, respectively. Among these hotspots, qualitative genes for stem rust resistance have been mapped on 3BS, 2BL and 5BL, while no qualitative genes have been characterized on 6BS. Clusters of QTLs were located distally on 5BS, 6BS and 7AS in regions where no previously reported qualitative or quantitative genes are located.

**Fig. 2:**
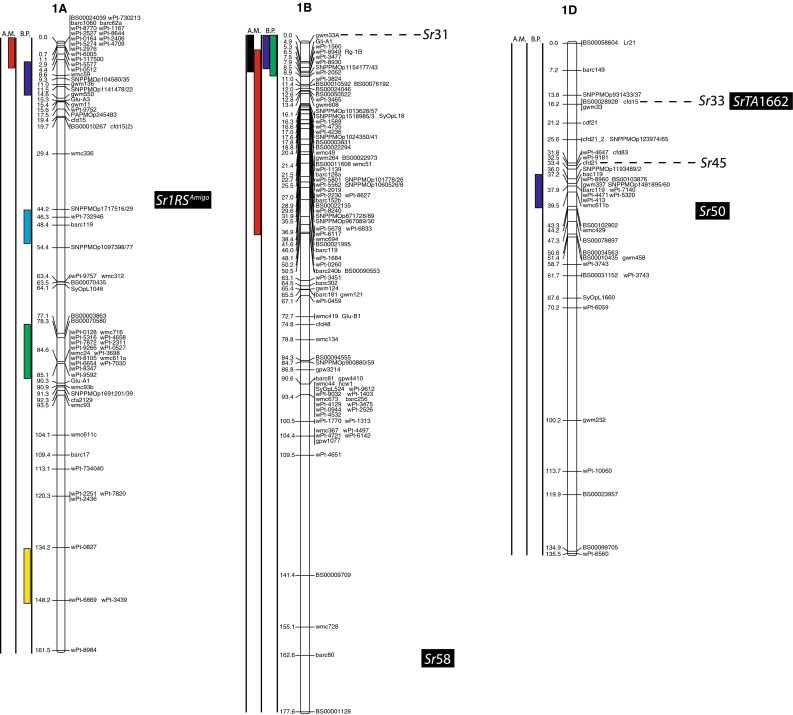

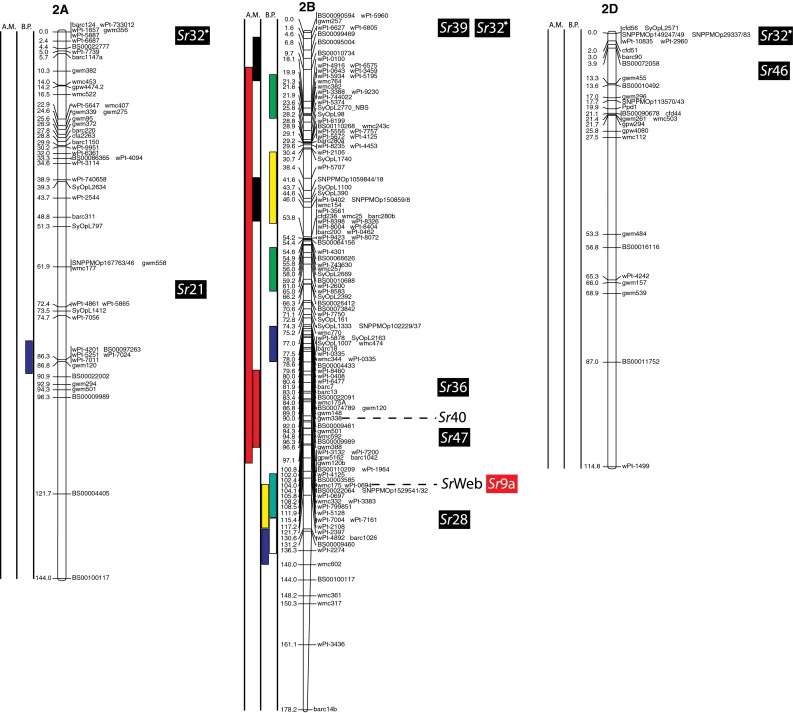

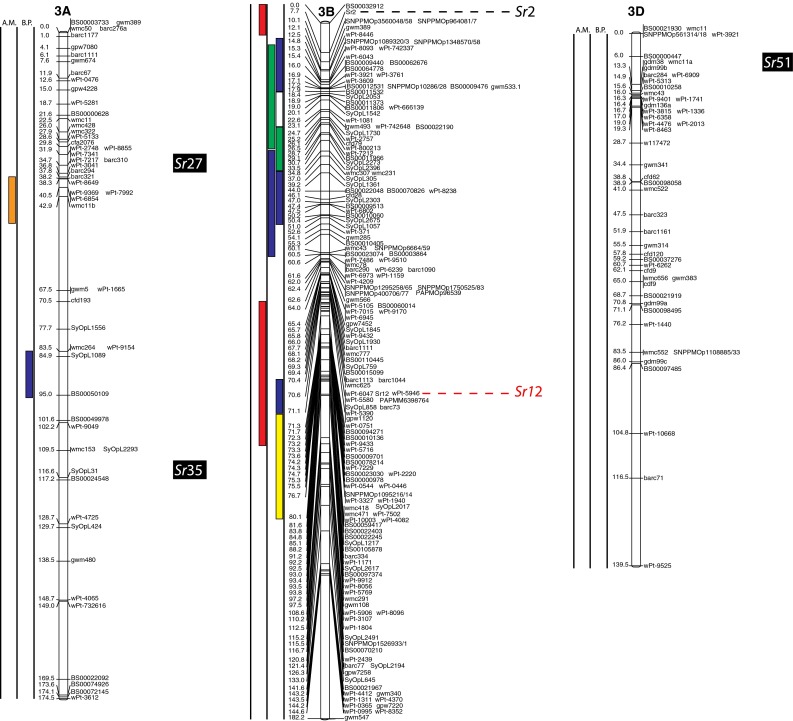

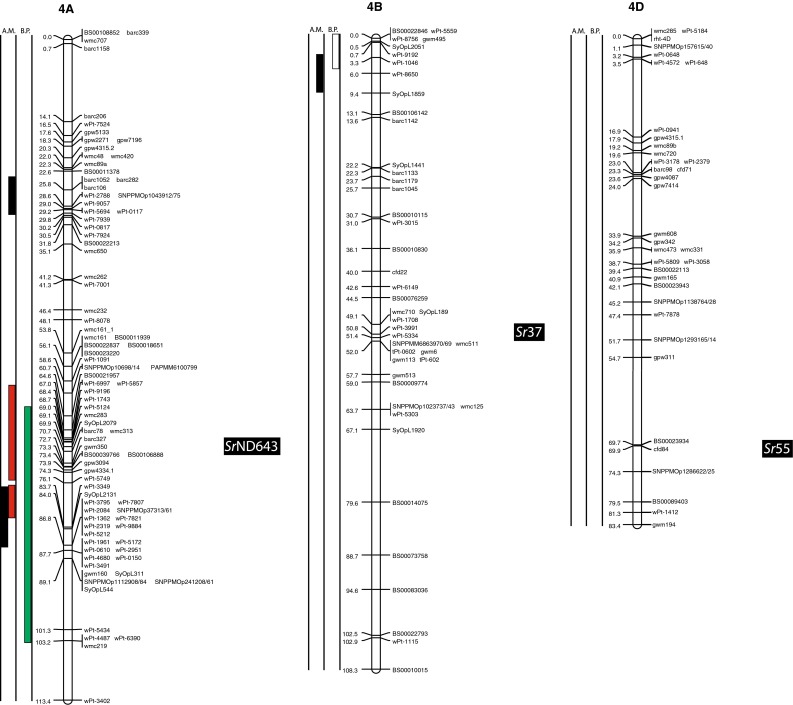

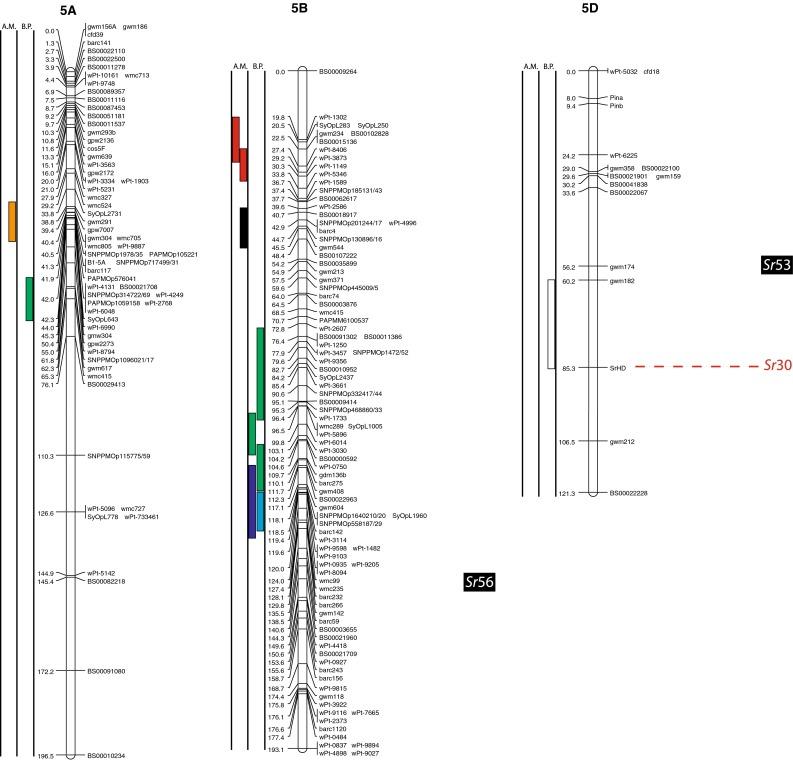

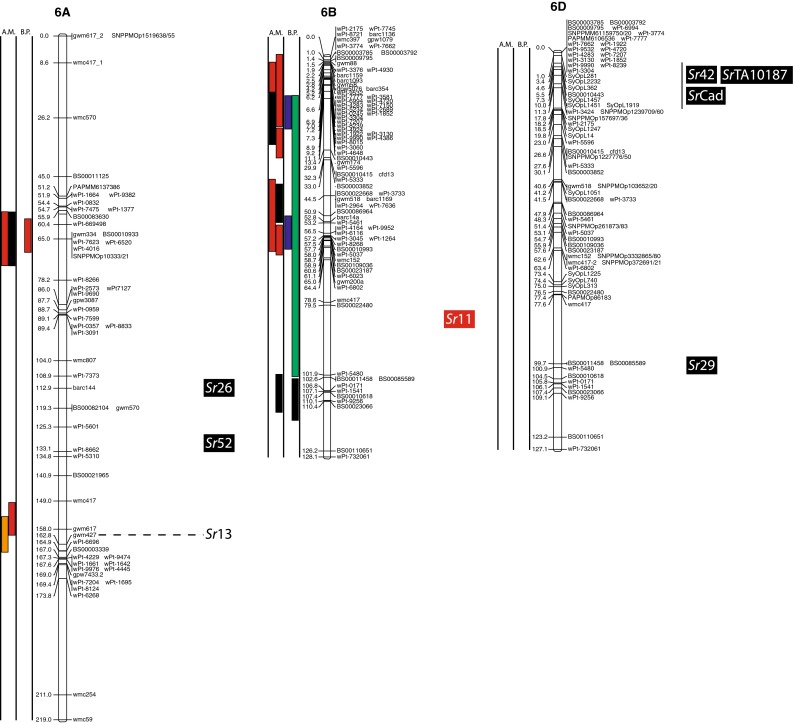

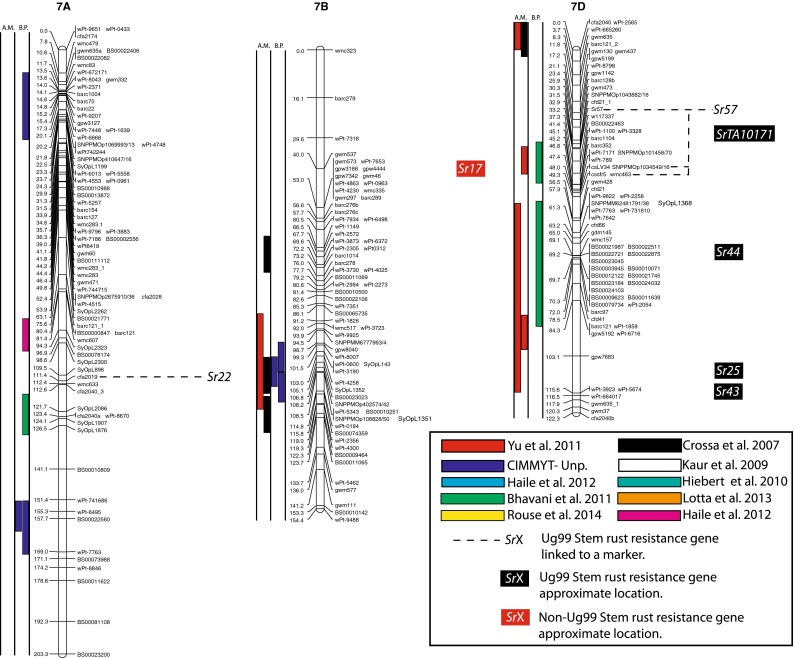
A consensus map of stem rust resistance loci in wheat. The map was constructed using the Wheat Interpolated Maps v4 (Diversity Arrays Technology Pty. Ltd., personal communication) as a reference map. DArT (prefix “wPt”), SNP (prefix “BS” or “SNP”) and SSR markers linked to stem rust resistance loci were integrated as described in the “Materials and methods”. The *bars with different colors or* *patterns* on the left side of chromosome regions distinguish mapping populations used in each study as shown in Table 1. Major genes that overlapped with QTLs were added to the right side of chromosomes based on the positions of the linked markers. *A.M.* association mapping, *B.P.* bi-parental population

Chromosome group 1 had 15 QTL and the cloned gene, *Sr33*, across the three homoeologous chromosomes. Six QTL were found on 1A (two on the short arm, three on the long arm and one is still in an undetermined arm location), five using bi-parental crosses (Thatcher/McNeal: Rouse et al. 2014; PBW343/Kingbird: Bhavani et al. 2011; PBW343/Kenya Swara; Kristal/Sebatel: Haile et al. 2012 and PBW343/Kenya Kudu: CIMMYT unpublished) and one using association mapping (LD-SRRSN (winter): Yu et al. 2012). Five of these QTL are represented in Fig. 2. One qualitative Ug99 resistance gene was mapped on 1A: *Sr1RS.* ^*Amigo*^ (Schlegel and Kynast 1987; Jin and Singh 2006; McIntosh et al. 2012). Seven QTL were found on 1B (three on the short arm, three on the long arm and one is still in an undetermined arm location). Four were mapped using bi-parental crosses (PBW343/Crossbill, PBW343/Kenya Nyangumi: CIMMYT unpublished; Avocet/Pavon76: Njau et al. 2013; Bhavani et al. 2011) and three using association mapping (ESWYT: Crossa et al. 2007; LD-SRRSN (spring): Yu et al. 2011 and the AM durum panel: Letta et al. 2013). Four of these QTL are represented in Fig. 2. *Sr31* (marker *XwPt*-*8949*) is on a 1BL.1RS rye translocation and is homologous with at least three of the QTL found in that region. *Sr14*, from tetraploid wheat origins (Heerman and Stoa 1956), was also reported on 1B, close to the centromere (McIntosh et al. 1995). The response of *Sr14* to Ug99 has been reported as inconclusive by Jin et al. (2007) and is not displayed in Fig. 2. Two QTL were mapped on chromosome 1D (one on the short arm and one in an undetermined arm location) using bi-parental crosses (HD2009/WL711: Kaur et al. 2009; Avocet/Pavon76: Njau et al. 2013). One of these QTL is represented in Fig. 2. The QTL linked to *XwPt*-*7140* on 1DS coincides with at least one of the four known *Sr* genes located in that region (*SrTA1662*, *Sr33*, *Sr45*, *Sr50*: Rouse et al. 2011; Sambasivam et al. 2008; Anugrahwati et al. 2008).

Chromosome group 2 had 19 QTL across the three homoeologous chromosomes. The short arm of chromosome 2A has two QTL where the centromeric stem rust resistance gene *Sr21* (The 1973) is located and the long arm had one QTL. One of these is represented in Fig. 2. Two *Sr* genes have been described on chromosome 2A, *Sr21* (The 1973), derived from *Triticum monococcum* L., as well as *Sr32* (McIntosh et al. 1995). It has to be noted that stem rust gene *Sr32* has also been mapped on chromosome 2B (McIntosh et al. 2011) as well as 2D (Mago et al. 2013). These three locations are displayed in Fig. 2, and *Sr32* is displayed distally to each chromosome 2 arm in Fig. 2. Chromosome 2B is the location of 15 QTL (two on the short arm, seven on the long arm and the arm location of six loci are unknown), 10 mapped in bi-parental crosses (Avocet/Pavon76: Njau et al. 2013; PBW343/Kenya Nyagumi and PBW343/Kenya Kudu: CIMMYT unpublished; PBW343/Juchi, PBW343/Huirivis#1, PBW343/Muu: Bhavani et al. 2011; HD2009/WL711: Kaur et al. 2009; RL6071/Webster: Hiebert et al. 2010; SD1691/LMPG-6: Rouse et al. 2012; Thatcher/McNeal: Rouse et al. 2014) and 5 detected using association mapping (ESWYT: Crossa et al. 2007; LD-SRRSN (spring); AM durum panel: Letta et al. 2013 and LD-SRRSN (winter): Yu et al. 2011). Twelve of these loci are shown in Fig. 2. There are 11 designated *Sr* genes on 2B (seven of them are shown in Fig. 2) in addition to Ug99-effective *SrWeb* (Hiebert et al. 2010). At least two QTL were identified on 2BS where seven *Sr* genes have been reported. Among them, *Sr39* (Niu et al. 2011), *Sr40* (Wu et al. 2008), and *Sr36* (Rouse et al. 2012) are effective against the original Ug99 race TTKSK and became ineffective to the variant TTTSK (Jin et al. 2009). Resistance genes *SrWeb*, *Sr28*, and *Sr47* have been reported on chromosome arm 2BL (Faris et al. 2008; Hiebert et al. 2011; Rouse et al. 2012). Chromosome 2D also had a single QTL in a bi-parental cross (PBW343/Kiritati: Bhavani et al. 2011; location not shown). Chromosome 2DS carries two qualitative *Sr* loci (*Sr32* and *Sr46;* Mago et al. 2013; E. Lagudah, personal communication, 2010) at the position of the QTL near marker *Xbarc90*.

On chromosome group 3, 26 QTL were reported as well as a cloned gene across the three homoeologous chromosomes. A single QTL was mapped on 3AS using association mapping (AM durum panel: Letta et al. 2013) and two QTL were mapped on 3AL, one using a bi-parental cross (PBW343/Kenya Kudu: CIMMYT unpublished) and one by association mapping (AM durum panel: Letta et al. 2013). Two of these QTL are shown in Fig. 2. Chromosome 3A carries two *Sr* genes, one cloned by Saintenac et al. (2013), *Sr35* on the long arm and the other one, *Sr27*, is located on the short arm (Acosta 1963). The other 23 QTL were on 3B (2 are on the long arm, 19 on the short arm and the locations of 2 are unknown). Eighteen were mapped using bi-parental crosses (Thatcher/McNeal: Rouse et al. 2014; PBW343/Crossbill, PBW343/Kenya Nyangumi, PBW343/Diniza, PBW343/Kenya Swara, PBW343/Kenya Kudu and PBW343/Pavon76: CIMMYT unpublished; HD2009/WL711: Kaur et al. 2009; PBW343/Kingbird, PBW343/Kiritaki, PBW343/Juchi, PBW343/Huirivis#1, PBW343/Muu and Avocet/Pavon76: Bhavani et al. 2011 and Kristal/Sebatel: Haile et al. 2012). Five were mapped by association analysis (ESWYT: Crossa et al. 2011; LD-SSRN (spring): Yu et al. 2011). Ten of the mapped loci are shown in Fig. 2. Eleven of the QTL on chromosome 3BS are coincident with the slow rusting gene, *Sr2*, that contributes to APR (Singh et al. 2006). QTL near the centromere of chromosome 3B appear to be linked to the seedling resistance gene *Sr12*. Though *Sr12* was not characterized as effective as Ug99 (Jin et al. 2007), recent data suggest that it interacts with other resistance loci to confer APR to Ug99 (Rouse et al. 2014).

Ten QTL were located on group 4 chromosomes. One QTL was located on 4AS, three were on 4AL and one was at an unknown location. Among them, one was identified using a bi-parental cross (PBW343/Juchi: Bhavani et al. 2011) and four using association mapping (LD-SRRSN (winter) and LD-SRRSN (spring): Yu et al. 2011; ESWYT: Crossa et al. 2007). Chromosome 4A carries one *Sr* gene temporarily designated as *SrND643* (CIMMYT unpublished), overlapping with QTL from several studies. Though Ug99 effective resistance gene *Sr37* has been introgressed into chromosome 4B, this translocation has not been used in a breeding program (McIntosh et al. 1995). We did not identify any QTL coincident with the previously described APR gene *Sr55* (*Lr67/Yr46/Pm46*) (McIntosh et al. 2012) on chromosome arm 4DL.

Group 5 had 18 QTL, 3 of which were on chromosome 5A. One of them was identified using a bi-parental cross (Avocet/Pavon76: Njau et al. 2013), and two by association mapping (AM durum panel: Letta et al. 2013). Two of these loci are shown in Fig. 2. Thirteen QTL were found on chromosome 5B, ten using bi-parental crosses (Arina/Forno: Bansal et al. 2008; PBW343/Kingbird, PBW343/Kiritaki, PBW343/Juchi, PBW343/Huirivis#1 and PBW343/Muu: Bhavani et al. 2011; PBW343/Kenya Kudu and PBW343/Kenya Nyangumi: CIMMYT unpublished; Kristal/Sebatel: Haile et al. 2012) and three using association mapping (ESWT: Crossa et al. 2007; LD-SSRN (Spring) and LD-SSRN (winter): Yu et al. 2011). Eight of the 5B QTL are shown in Fig. 2. Kaur et al. (2009) detected a distal 5BS QTL in three of the four environments tested using a bi-parental population. This finding was confirmed by Yu et al. (2011) in the detection of two QTL distally located on chromosome 5BS by association mapping in spring and winter CIMMYT germplasm between markers *XwPt1149*/*XwPt 5346* and *XwPt1302*/*XwPt3873*, respectively. Both QTL were minor but overlapped with the QTL detected by Kaur et al. (2009) (marker *wPt5346*). The QTL on 5BL from the Arina/Forno population was recently designated as *Sr56* (McIntosh et al. 2012) and confers APR. No other stem rust resistance genes have been designated on chromosome 5B. Ug99 resistance gene *Sr53* was recently introgressed from *Aegilops geniculata* to chromosome arm 5DL where two Ug99 QTL were identified using bi-parental crosses (HD2009/WL711: Kaur et al. 2009; RL6071/Webster: Hiebert et al. 2010).

Group 6 had 23 QTL across the three homoeologous chromosomes. On chromosome 6A two QTL were on the short arm and six on the long arm. Two were mapped using a bi-parental cross (PBW343/Kenya Swara: CIMMYT unpublished; Kristal/Sebatel: Haile et al. 2012) and six using association mapping (ESWT: Crossa et al. 2007; LD-SRRSN (spring): Yu et al. 2011; AM durum panel: Letta et al. 2013). Five of the 6A loci are represented in Fig. 2. Three designated *Sr* genes are located on 6A, including three Ug99 resistance genes located on chromosome arm 6AL (*Sr26*, *Sr13*, and *Sr52;* Hart et al. 1993; McIntosh 1972; Qi et al. 2011). *Sr52* is not currently used in breeding programs. On 6B there were 13 QTL (nine on the short arm, two on the long arm and two have an unknown arm location), 5 were mapped in bi-parental crosses (PBW343/Juchi, Avocet/Pavon76: Bhavani et al. 2011; PBW343/Crossbill, PBW343/Kenya Nyangumi: CIMMYT unpublished; Norin40/LMPG-6: Ghazvini et al. 2012) and 8 by association analysis (LD-SRRSN (spring) and LD-SRRSN (winter): Yu et al. 2011; ESWYT: Crossa et al. 2007). Twelve of these QTL are represented in Fig. 2. No major Ug99 resistance genes are known to be located in this region. The 6DS chromosome arm had one resistance gene identified in a BC_2_ population (*SrTA1662*; BC_2_-TA10187/KS05HW14: Olson et al. 2013) and a QTL identified in bi-parental crosses (AC Cadillac/Carberry: Lopez-Vera et al. 2014). Neither of the 6DS loci are shown in Fig. 2 because of difficulty in cross referencing the chromosome location. Four Ug99 resistance *Sr* genes have been identified on chromosome 6D. Three of them are located on the short arm, *Sr42*, *SrTA10187* and *SrCad* (Hiebert et al. 2011; Ghazvini et al. 2012; Lopez-Vera et al. 2014) and one on the long arm, *Sr29* (Dyck and Kerber 1977).

Group 7 had 30 QTL across the three homoeologous chromosomes. Three were mapped on 7AS, three on 7AL, and three have unknown locations. Seven were mapped in bi-parental populations (HD2009/WL711: Kaur et al. 2009; PBW343/Kingbird: Bhavani et al. 2011; PBW343/Diniza, PBW343/Kenya Swara, PBW343/Kenya Nyangumi: CIMMYT, unpublished; Kristal/Sebatel: Haile et al. 2012) and two were mapped by association mapping (AM durum panel: Letta et al. 2013). Five of these QTL are shown in Fig. 2. Gene *Sr15* (not shown in Fig. 2) and Ug99 effective gene *Sr22* (The 1973) are located on 7AL. On 7B there were ten QTL, five of which were mapped using bi-parental crosses (PBW343/Huirivis#1: Bhavani et al. 2011; PBW343/Kenya Nyangumi, PBW343/Kenya Kudu: CIMMYT, unpublished; Avocet/Pavon76: Njau et al. 2013; Kristal/Sebatel: Haile et al. 2012) and five using association analysis (ESWYT: Crossa et al. 2007; LD-SRRSN (spring) and LD-SRRSN (winter): Yu et al. 2011). Seven of these QTL are represented in Fig. 2. Eight of these QTL are mapped near *Sr17* (position not represented in Fig. 2) (Bansal et al. 2008). The 7D chromosome had 11 QTL, 6 mapped in bi-parental crosses (Thatcher/McNeal: Rouse et al. 2014; PBW343/Kiritaki, PBW343/Kingbird: Bhavani et al. 2011; Arina/Forno: Bansal et al. 2008; PBW343/Kenya Swara: CIMMYT, unpublished; BC2-TA10171/KS05HW14: Olson et al. 2013), whereas five were mapped in association panels (ESWYT: Crossa et al. 2007; LD-SRRSN (spring) and LDSRRSN (winter): Yu et al. 2011). Seven of these QTL are shown in Fig. 2. Pleiotropic rust and powdery mildew resistance gene *Sr57* (*Lr34*/*Yr18*/*Pm38*), *Sr44* as well as *SrTA10171* are located on 7DS (Kolmer et al. 2011; Bernd Friebe, personal communication) and *Sr25* as well as *Sr43* (Xu et al. personal communication) are located on 7DL.

## Discussion

Four maps including the Wheat Interpolated DArT Maps v4, the wheat consensus SSR map, the wheat KASPar SNP map, and the Thatcher/McNeal DArT/SSR markers map were used to build the consensus map for locating the stem rust resistance loci (Fig. 2). The consensus map consisted of 1,433 markers and an average marker interval of 11.5 cM. We focused on integrating markers in the regions spanning stem rust resistance QTL that can further facilitate fine mapping and cross referencing the locations with other maps. Therefore, the marker density in the QTL regions is higher than the average. For example, marker density is almost three times higher in the QTL region of rust resistance genes on 1B (Fig. 2, Chromosome 1B). The same is true for 2BL, 3BS, 4AL, 6BS and 7DL. However, the complexity and context dependency of QTL identified in different genetic backgrounds and environments can limit the accuracy of the locations. The accurate genome location of QTL and major genes across genetic backgrounds and environments is a prerequisite for the use of the QTL in MAS. Meta-analysis of QTL identified in different studies can locate QTL more precisely, thus facilitating the identification of closely linked markers for MAS. Because many of the populations from which APR was assessed lacked Ug99-effective qualitative resistance genes, the coincidence of APR with seedling resistance was not likely to be a result of the qualitative resistance genes conferring APR. For example, on 2BS, seven QTL were identified that overlapped with two Ug99-effective stem rust resistance genes, *Sr39* and *Sr36* (Fig. 2b). Since *Sr39* was not present in the wheat cultivars used to map the QTL and *Sr36* was only present in the germplasm analyzed by Yu et al. (2012) where no association was found between *Sr36* and APR, the QTL on 2BS could be conferred by alleles of these qualitative genes, residual effects of other *Sr* genes on 2BS, or new genes. Similarly, the stem rust resistance gene *Sr40* on 2BS is strongly associated with marker *Xgwm388* and coincided with one QTL, however *Sr40* was not present in the corresponding germplasm (Yu et al. 2011). Allelism testing utilizing both adult plant and seedling testing to identify both qualitative and quantitative resistance loci will be necessary to sort out the allelic relationships among many of the QTL and *Sr* genes reported on wheat chromosomes.

In spite of the complexity of the meta-QTL analysis, using the available information on the *Sr* genes in the parents of biparental populations and accessions used for association mapping combined with the QTL location based on anchored markers, we were able to identify (a) QTL underlying some previously described Ug99 resistant *Sr* genes or residual effects of non Ug99 resistant genes and (b) putative locations of new *Sr* genes.

### Colocation of Sr genes and QTL

Even though *Sr31* carried by the rye introgression 1BL.1RS is not effective to Ug99, the three QTL found on chromosome 1BS, homoeologous to 1RS, are possibly due to a residual effect of *Sr31* or another gene on the rye translocation because wheat cultivar PBW343 possesses *Sr31*. Additional studies are needed to validate whether the 1BS QTL are effects of the 1BL.1RS translocation or if there are one or more new APR genes on 1BS. Adult plant resistance gene *Sr58* (*Lr46/Yr29/Pm39*) mapped distally on 1BL (McIntosh et al. 2012) and is independent of the seven QTL on chromosome 1BS. The response of *Sr14* to Ug99 (Jin et al. 2007) was inconclusive, but because of its tetraploid origin and linkage to centromeric markers, the QTL on 1BS highlighted by Letta et al. (2013) is likely conferred by *Sr14*. Chromosome arm 2B, with 15 QTL, has the second highest number of QTL per chromosome arm, but also the highest number of known qualitative Ug99 *Sr* genes (6 numerically designated: *Sr39*, *Sr32*, *Sr36*, *Sr40*, *Sr47*, *Sr28* and *SrWeb*). Sorting out the allelic relationships among these QTL and at least 11 qualitative *Sr* genes (seven Ug99 resistant and four non Ug99 resistant *Sr* genes) on chromosome 2B will be necessary to determine whether any new *Sr* genes have been detected or if they are the results of residual effects. The three QTL found in the AM durum panel (Letta et al. 2013) on chromosome 6AL overlap with *Sr13*, and even though *Sr13* is not common in bread wheat, its presence in durum wheat suggests that the large region highlighted is most likely *Sr13*. Similarly, further studies are required to determine the allelic relationships among *SrTA1662*, *SrCad*, and *Sr42* on 6DS (Hiebert et al. 2011; Ghazvini et al. 2012; Lopez-Vera et al. 2014). Chromosome 7B has nine QTL, eight of these QTL mapped near *Sr17* (position not represented on Fig. 2) (Bansal et al. 2008). Though *Sr17* is not effective to Ug99 in a qualitative manner (Jin et al. 2007) it is possible that *Sr17* confers a residual APR effect. On chromosome 7D, early studies indicated that *Sr57* (*Lr34*/*Yr18*/*Pm38*), *Sr58* (*Lr46/Yr29/Pm39*) enhanced stem rust resistance in cultivar ‘Thatcher’ (Dyck and Kerber 1977; Kerber and Aung 1999), and later reports suggested that *Sr57* provided APR to stem rust in diverse backgrounds (McIntosh et al. 2012). Our previous studies consistently showed that the STS marker csLV34 was significantly associated with Ug99 resistance in winter and spring CIMMYT wheat panels with major effects or through gene–gene interactions (Yu et al. 2011, 2012). Overlap of two QTL located distally on 7DL (Yu et al. 2011) are likely conferred by *Sr25* (Ayala-Navarrete et al. 2007) because *Sr43* and *Sr44* are not currently used in wheat breeding.

### Putative locations of new *Sr* genes

With six QTL found on 1A, that chromosome could be a potential new source of new APR. Because *Sr1RS* ^*Amigo*^ was introgressed from rye and confers qualitative resistance, it is most likely different from the six QTL on 1A. The same is true for the two QTL found on chromosome 1D. Even though they overlap with four described Ug99 resistant *Sr* genes (*SrTA1662*, *Sr33*, *Sr45*, *Sr50*: Rouse et al. 2011; Sambasivam et al. 2008; Anugrahwati et al. 2008), the *Aegilops tauschii* or rye origin of these genes make it unlikely that these QTL are related. The two QTL found on chromosome 2A are likely to represent new Ug99 *Sr* resistance loci because the only known gene in the region, *Sr21* (The 1973), is derived from *T. monococcum*, and because the parents of the population with the 2A QTL (PBW343/Pavon76: CIMMYT unpublished) are not known to possess *T. monococcum* in their pedigrees. Also, *Sr21* was not in the pedigrees of the AM durum panel (Letta et al. 2013). Chromosome group 3 has the second highest number of QTL, and chromosome 3B, with 23 QTL has the highest number of QTL by chromosome arm (Fig. 1). Chromosome arm 3BS has only one Ug99 *Sr* resistant gene, the slow rusting gene *Sr2* and 19 of the QTL found on that arm are likely *Sr2*. In the Thatcher/McNeal population, a QTL was coincident with *Sr12* on 3BL, a Ug99-ineffective resistance gene. It is possible that such defeated resistance genes could confer resistance when combined with genes such as *Sr57* (Lr34/Yr18/Pm38) that have been demonstrated to confer epistatic resistance to stem rust (Kolmer et al. 2011; Rouse et al. 2014; Yu et al. 2012). Because *Sr12* is present in the cultivar Thatcher and historically was used as an important source of resistance, *Sr12* could be widespread in wheat germplasm. Since no resistance genes have been characterized on 3BL, the QTL in this region are conferred by one or more new resistance genes. Ug99 resistance gene *Sr51* has been introgressed into translocations on each of the group 3 homoeologous chromosomes (Liu et al. 2011b). Since this gene is derived from *Aegilops searsii* and is not currently used in agriculture, none of the group 3 QTL are conferred by this gene. Chromosome 3A has three QTL, and the *Sr35* cloned gene seems to overlap with the QTL in a CIMMYT population (CIMMYT unpublished) and in the durum panel (Letta et al. 2013) on chromosome 3AL, these QTL are not *Sr35* because seedling resistance to race TTKSK is absent in the mapping population used by CIMMYT. In addition, the *T. monococcum* origin of *Sr35* is absent from the pedigrees of the panel used by Letta et al. (2013). Resistance gene *Sr27* is also on 3A, but this gene is different from these QTL because it is located on a rye introgression not present in the population used to identify these QTL. On Chromosome 4A, the stem rust resistance gene designated *SrND643* (CIMMYT unpublished) is a qualitative gene and the PBW343/Juchi population does not possess that resistance gene so the QTL from PBW343/Juchi is likely conferred by a new resistance gene or allele. The relationships among the QTL identified on 4AL, *SrND643*, and *Sr7* (Singh et al. 2006; Sears 1954) are not known. The distal end of chromosome 4B appears to be the source of a new *Sr* gene, although the variance explained for the QTL is low (9 %) and may represent a minor effect APR gene. Chromosome 5A does not carry any previously characterized *Sr* genes, so the three QTL identified on that chromosome arm likely represent new resistance loci. Although the phenotypic variation for the two QTL identified by Letta et al. (2013) on durum wheat is small (*R* ^2^ = 4.1 and 4.4 %), further studies of that region could support the existence of new Ug99 resistant loci. The APR gene *Sr56* (McIntosh et al. 2012) on chromosome 5BL was previously designated *QSr.Sun*-*5BL* (Bansal et al. 2008) (not represented in Fig. 2). Among the 12 other QTL found on 5B, at least four are located on the short arm (Bansal et al. 2008; CIMMYT unpublished; Kaur et al. 2009; Yu et al. 2011) and likely represent new loci of importance. Although Kaur et al. (2009) did not test for APR to Ug99, Yu et al. (2011) also mapped distal 5BS QTL providing strong evidence for minor APR genes located in this region. Resistance gene *Sr26* on 6AL is present in conventional common wheat germplasm from Australia, but was not present in CIMMYT germplasm when these studies were conducted. Therefore *Sr26* does not explain the QTL identified through association mapping (Crossa et al. 2007; Yu et al. 2011). A putative new resistance QTL on 6AS was mapped in the hexaploid biparental PBW343/Kenya Swara population where no qualitative resistance genes are located (CIMMYT, unpublished). The QTL found on 6AL in the tetraploid population Kristal/Sebatel is likely a new source of resistance since *Sr13*-linked markers are in the distal region of 6AL. Chromosome 6B seems to be a rich source of new *Sr* genes with nine QTL detected. The relationship between the QTL on 6BL and *Sr11* is not known but *Sr11* is ineffective against Ug99 and is not represented on Fig. 2. Chromosome arm 6BS appears to be a new source of *Sr* genes (Crossa et al. 2007; Yu et al. 2011; CIMMYT unpublished). The phenotypic variation explained by these QTL ranged from 5 to 14 % and most likely correspond to minor APR genes. Singh et al. (2011) detected a distal QTL that explained 56 % of the variance for stem rust on chromosome 6BS (XwPt4283/XwPt7207) in the bi-parental cross between PBW343/Kenya Nyangumi. Validation of that QTL could be accomplished using the putative allele carrier, Kenya Nyangumi, in another bi-parental cross. The QTL on 7AS, either from durum or bread wheat, once validated, would represent new stem rust resistance genes as *Sr22* is located on 7AL. Since *Sr22* was not present in the parents of the hexaploid mapping populations, these QTL are not conferred by *Sr22*. The more distal QTL found in the Kristal/Sebatel bi-parental tetraploid population (Q*Sr.1PK*-*7A.2*) is likely to be conferred by *Sr22* (Haile et al. 2012). Since *Sr* genes have not been previously identified on 7BS, the QTL identified in this region are likely new.

Overall, the growing number of characterized *Sr* genes and QTL demonstrates that there is still potential for discovering new APR genes with varying levels of effect. This consensus map will facilitate the identification of new resistance genes and QTL and aid in the development of improved markers to increase breeding efficiency and the pool of alleles that are important for the control of Ug99.

## Electronic supplementary material

Below is the link to the electronic supplementary material.

Supplementary material 1 (PDF 560 kb)
