# Supplementary material for: A consensus map for Ug99 stem rust resistance loci in wheat
Source: Theor Appl Genet. 2014 Jun 6;127(7):1561–81. doi: 10.1007/s00122-014-2326-7 (PMC4072096; doi:10.1007/s00122-014-2326-7)
Supplement: Supplementary file 1 — Supplementary material 1 (PDF 560 kb) [file 122_2014_2326_MOESM1_ESM.pdf]

Chromosome 1A

1	BS00024039	0
2	wPt-730213	0
3	barc1060	0
4	barc62a	0
5	wPt-8770	0
6	wPt-1167	0
7	wPt-2527	0
8	wPt-8644	0
9	wPt-0164	0
10	wPt-2406	0
11	wPt-5274	0
12	wPt-4709	0
13	wPt-2976	0
14	wPt-6005	0.74
15	w117500	1.13
16	wPt-5577	2.89
17	wPt-0512	4.41
18	wmc59	8.56
19	SNPPMOp104580/35	9.33
20	gwm136	11.01
21	SNPPMOp1141478/22	11.53
22	gwm550	14.59
23	Glu-A3	15.27
24	gwm11	15.43
25	wPt-9752	15.63
26	PAPMOp245483	17.53
27	cfd15	19.43
28	BS00010267	19.65
29	cfd15(2)	19.65
30	wmc336	29.43
31	SNPPMOp1717516/29	44.23
32	wPt-732946	46.34
33	barc119	48.43
34	SNPPMOp1097398/77	54.43
35	wPt-9757	63.43
36	wmc312	63.43
37	BS00070435	63.45
38	SyOpL1046	64.13
39	BS00003863	77.09
40	BS00070580	78.27
41	wPt-0128	84.61
42	wmc716	84.61
43	wPt-5316	84.61
44	wPt-4658	84.61
45	wPt-7872	84.61
46	wPt-2311	84.61
47	wPt-9266	84.61
48	wPt-0527	84.61
49	wmc24	84.61
50	wPt-3698	84.61
51	wPt-8105	84.61
52	wmc611a	84.61
53	wPt-6654	84.61
54	wPt-7030	84.61
55	wPt-8347	84.61
56	wPt-9592	85.09
57	Glu-A1	90.26
58	wmc93b	90.93
59	SNPPMOp1691201/39	91.33
60	cfa2129	92.28
61	wmc93	93.52
62	wmc611c	104.13
63	barc17	109.43
64	wPt-734040	113.05
65	wPt-2251	120.26
66	wPt-7820	120.26
67	wPt-2436	120.26
68	wPt-0827	134.19
69	wPt-6869	148.18
70	wPt-3439	148.18
71	wPt-8984	161.47

Chromosome 1B

1	gwm33A	0
2	Gli-A1	4.88
3	wPt-1560	5.28
4	wPt-8949	6.51
5	Rg-1B	6.53
6	wPt-3477	7.53
7	wPt-8930	7.86
8	SNPPMOp1154177/43	8.48
9	wPt-2052	8.88
10	wPt-3824	11.02
11	BS00010592	11.37
12	BS00076192	11.37
13	BS00024046	12.02
14	BS00050522	12.59
15	wPt-3465	12.81
16	gwm608	13.38
17	SNPPMOp1013628/57	16.08
18	SyOpL18	16.08
19	SNPPMOp1518985/3	16.08
20	wPt-1589	16.28
21	wPt-4735	16.57
22	wPt-4236	17.01
23	SNPPMOp1024350/41	17.58
24	BS00003831	17.75
25	BS00022294	18.81
26	wmc49	20.38
27	gwm264	21.38
28	BS00022973	21.38
29	BS00011608	21.38
30	wmc51	21.38
31	wPt-1139	21.38
32	barc128a	21.5
33	wPt-5801	22.68
34	SNPPMOp101778/26	22.68
35	wPt-5562	25.48
36	SNPPMOp1060526/8	25.48
37	wPt-2019	25.48
38	wPt-2230	26.96
39	wPt-8627	26.96
40	barc152b	26.96
41	BS00022135	28.89
42	wPt-8240	29.57
43	SNPPMOp871728/89	31.88
44	SNPPMOp967089/30	35.48
45	wPt-5678	36.85
46	wPt-6833	36.85
47	wPt-6117	36.85
48	wmc694	38.38
49	BS00021995	41.55
50	barc119	46.03
51	wPt-1684	48.13
52	wPt-0260	50.24
53	barc240b	50.47
54	BS00090553	50.47
55	wPt-3451	63.12
56	barc302	64.47
57	gwm124	65.38
58	barc181	65.5
59	gwm121	65.5
60	wPt-0459	67.08
61	wmc419	72.69
62	Glu-B1	72.69
63	cfd48	74.82
64	wmc134	78.79
65	BS00094555	84.32
66	SNPPMOp900880/59	84.68
67	gpw3214	86.79
68	barc81	90.59
69	gpw4410	90.59
70	wmc44	93.38
71	ncw1	93.38
72	SyOpL524	93.38
73	wPt-9612	93.38
74	wPt-9032	93.38
75	wPt-1403	93.38
76	wmc673	93.38
77	barc256	93.38
78	wPt-4129	93.38
79	wPt-3475	93.38
80	wPt-0944	93.38
81	wPt-2526	93.38
82	wPt-4532	93.38
83	wPt-1770	100.51
84	wPt-1313	100.51
85	wmc367	104.38
86	wPt-4497	104.38
87	wPt-4721	104.38
88	wPt-6142	104.38
89	gpw1077	104.38
90	wPt-4651	109.49
91	BS00009709	141.36
92	wmc728	155.12
93	barc80	162.55
94	BS00001128	177.63

Chromosome 1D

1	BS00058604	0
2	Lr21	0
3	barc149	7.22
4	SNPPMOp931433/37	13.83
5	BS00028928	16.22
6	cfd15	16.22
7	gwm33	16.22
8	cdf21	21.22
9	cfd21_2	25.63
10	SNPPMOp123974/65	25.63
11	wPt-4647	31.84
12	cfd83	31.84
13	wPt-9181	32.53
14	cfd21	33.4
15	SNPPMOp1193489/2	36.03
16	bac119	37.22
17	wPt-8960	37.93
18	BS00103876	37.93
19	gwm337	37.93
20	SNPPMOp1481895/60	37.93
21	barc119	37.93
22	wPt-7140	37.93
23	rPt-4471	37.93
24	wPt-5320	37.93
25	wPt-413	37.93
26	wmc611b	39.53
27	BS00102902	43.31
28	wmc429	44.22
29	BS00078897	47.29
30	BS00034563	50.56
31	BS00010435	51.37
32	gwm458	51.37
33	wPt3743	58.69
34	BS00031152	61.74
35	wPt-3743	61.74
36	SyOpL1660	67.63
37	wPt-6059	70.16
38	gwm232	100.22
39	wPt-10060	113.73
40	BS00023957	119.85
41	BS00099705	134.86
42	wPt-6560	135.47

Chromosome 2A

1	barc124	0
2	wPt-733012	0
3	wPt-1657	0
4	gwm356	0
5	wPt-5887	0
6	wPt-6687	2.36
7	BS00022777	4.36
8	wPt-7739	4.96
9	barc1147a	5.74
10	gwm382	10.33
11	wmc453	13.95
12	gpw4474.2	14.24
13	wmc522	16.54
14	wPt-5647	22.86
15	wmc407	22.86
16	gwm339	24.61
17	gwm275	24.61
18	gwm95	25.58
19	gwm372	26.86
20	barc220	27.83
21	cfa2263	28.81
22	barc1150	29.86
23	wPt-9951	30.21
24	wPt-6361	31.96
25	BS00086365	33.32
26	wPt-4094	33.32
27	wPt-3114	34.56
28	wPt-740658	38.88
29	SyOpL2634	39.26
30	wPt-2544	43.7
31	barc311	48.81
32	SyOpL797	51.26
33	SNPPMOp167763/46	61.86
34	gwm558	61.86
35	wmc177	61.86
36	wPt-4861	72.36
37	wPt-5865	72.36
38	SyOpL1412	73.46
39	wPt-7056	74.73
40	wPt-4201	86.27
41	BS00097263	86.27
42	wPt-5251	86.27
43	wPt-7024	86.27
44	wPt-7011	86.27
45	gwm120	86.77
46	BS00022002	90.86
47	gwm294	92.86
48	gwm501	94.28
49	BS00009989	96.27
50	BS00004405	121.68
51	BS00100117	144.04

Chromosome 2B

1	BS00090594	0
2	wPt-5960	0
3	gwm257	0
4	wPt-6627	1.61
5	wPt-6805	1.61
6	BS00099469	4.59
7	BS00095004	6.82
8	BS00010734	9.73
9	wPt-0100	18.1
10	wPt-4916	19.92
11	wPt-6575	19.92
12	wPt-0643	19.92
13	wPt-3459	19.92
14	wPt-5934	19.92
15	wPt-5195	19.92
16	wmc764	21.26
17	wmc382	21.57
18	wPt-3388	21.9
19	wPt-9230	21.9
20	wPt-744022	21.9
21	wPt-5374	23.6
22	SyOpL2770_NBS	25.8
23	SyOpL98	28.2
24	wPt-6199	28.81
25	BS00110268	28.91
26	wmc243c	28.91
27	wPt-5556	29.12
28	wPt-7757	29.12
29	wPt-5672	29.12
30	wPt-4125	29.12
31	barc280a	29.24
32	wPt-8235	29.58
33	wPt-4453	29.58
34	wPt-2106	30.35
35	SyOpL1740	30.7
36	wPt-5707	38.4
37	SNPPMOp1059844/18	41.6
38	SyOpL1100	43.7
39	SyOpL390	44.6
40	wPt-9402	46
41	SNPPMOp150859/8	46
42	wmc154	46
43	wPt-3561	53.8
44	barc280b	53.8
45	cfd238	53.8
46	wmc25	53.8
47	wPt-8398	53.8
48	wPt-8326	53.8
49	wPt-8004	53.8
50	wPt-8404	53.8
51	barc200	53.8
52	wPt-0462	53.8
53	wPt-9423	54.17
54	wPt-8072	54.17
55	BS00064156	54.37
56	wPt-4301	54.57
57	BS00066626	54.92
58	wPt-743630	55.78
59	wmc257	56
60	SyOpL2669	58
61	BS00010688	59.17
62	wPt-2600	61
63	wPt-8583	65.02
64	SyOpL2392	66.2
65	BS00026412	66.33
66	BS00073842	70.6
67	wPt-7750	71.09
68	SyOpL161	72.8
69	SyOpL1333	74.3
70	SNPPMOp102229/37	74.3
71	wmc770	75.16
72	wPt-5878	77
73	SyOpL2163	77
74	SyOpL1007	77
75	wmc474	77
76	barc18	77
77	wPt-0335	77.5
78	wmc344	78
79	wPt0335	78
80	BS00004433	78.64
81	wPt-8460	79.55
82	wPt-0408	80.03
83	wPt-6477	80.35
84	barc7	81.86
85	barc13	83
86	BS00022091	83.4
87	wmc175A	83.96
88	BS00074789	86.77
89	gwm120	86.77
90	gwm148	89.02
91	gwm338	90
92	BS00009461	91.96
93	gwm501	94.28
94	wmc592	94.75
95	BS00009989	96.27
96	gwm388	96.62
97	wPt-3132	97.1
98	wPt-7200	97.1
99	gpw5162	97.1
100	barc1042	97.1
101	gwm120b	97.1
102	BS00110209	100.77
103	wPt-1964	100.8
104	tPt-4125	101.95
105	BS00003585	102.43
106	wmc175	104
107	wPt-0694	104.04
108	BS00022064	104.1
109	SNPPMOp1529541/32	104.1
110	wPt-0697	105.82
111	wmc332	108.16
112	wPt-3383	108.16
113	wPt-799851	108.46
114	wPt-5128	111.88
115	wPt-7004	115.44
116	wPt-7161	115.44
117	wPt-2108	117.19
118	wPt-2397	121.7
119	wPt-4892	130.61
120	barc1026	130.61
121	BS00009460	131.2
122	wPt-2274	136.3
123	wmc602	140
124	BS00100117	144.04
125	wmc361	148.16
126	wmc317	150.32
127	wPt-3436	161.06
128	barc14b	178.19

Chromosome 2D

1	cfd56	0
2	SyOpL2571	0
3	SNPPMOp149247/49	0
4	SNPPMOp29337/83	0
5	wPt-10835	0
6	wPt-2960	0
7	cfd51	2
8	barc90	3
9	BS00072058	3.85
10	gwm455	13.31
11	BS00010492	13.56
12	gwm296	17
13	SNPPMOp113570/43	17.7
14	Ppd1	19.92
15	BS00090678	21.13
16	cfd44	21.13
17	gwm261	21.35
18	wmc503	21.35
19	gpw294	21.68
20	gpw4080	25.76
21	wmc112	27.51
22	gwm484	53.32
23	BS00016116	56.79
24	wPt-4242	65.29
25	gwm157	66
26	gwm539	68.9
27	BS00011752	87.01
28	wPt-1499	114.78

Chromosome 3A

1	BS00003733	0
2	gwm389	0
3	wmc50	0
4	barc276a	0
5	barc1177	0.97
6	gpw7080	4.09
7	barc1111	6.06
8	gwm674	7.63
9	barc67	11.87
10	wPt-0476	12.62
11	gpw4228	15.04
12	wPt-5281	18.67
13	BS00000628	21.57
14	wmc11	22.53
15	wmc428	25.98
16	wmc322	27.92
17	wPt-5133	28.6
18	cfa2076	29.75
19	wPt-2748	31.92
20	wPt-8855	31.92
21	wPt-7341	31.92
22	wPt-7217	34.7
23	barc310	34.7
24	wPt-3041	36.8
25	barc294	37.83
26	barc321	38.15
27	wPt-8649	38.25
28	wPt-9369	40.51
29	wPt-7992	40.51
30	wPt-6854	40.51
31	wmc11b	42.93
32	gwm5	67.53
33	wPt-1665	67.53
34	cfd193	70.53
35	SyOpL1556	77.73
36	wmc264	83.53
37	wPt-9154	83.53
38	SyOpL1089	84.93
39	BS00050109	95.02
40	BS00049978	101.64
41	wPt-9049	102.15
42	wmc153	109.53
43	SyOpL2293	109.53
44	SyOpL31	116.63
45	BS00024548	117.16
46	wPt-4725	128.7
47	SyOpL424	129.73
48	gwm480	138.53
49	wPt-4065	148.7
50	wPt-732616	149
51	BS00022092	169.52
52	BS00074926	173.58
53	BS00072145	174.13
54	wPt-3612	174.47

Chromosome 3B

1	BS00032912	0
2	Sr2	7.68
3	SNPPMOp3560048/58	10.05
4	SNPPMOp964081/7	10.05
5	gwm389	12.11
6	wPt-8446	12.51
7	SNPPMOp1089320/3	14.78
8	SNPPMOp1348570/58	14.78
9	wPt-8093	15.32
10	wPt-742337	15.32
11	wPt-6043	15.41
12	BS00009440	16.03
13	BS00062676	16.03
14	BS00064778	16.03
15	wPt-3921	16.87
16	wPt-3761	16.87
17	wPt-3609	16.91
18	gwm533.1	17.11
19	BS00009476	17.22
20	BS00012531	17.22
21	SNPPMOp10286/28	17.88
22	BS00011532	18.38
23	SyOpL2053	18.88
24	BS00011373	18.96
25	BS00011806	18.96
26	wPt-666139	20.12
27	SyOpL1542	22.58
28	wPt-1081	23.11
29	BS00022190	23.11
30	gwm493	23.11
31	wPt-742648	23.11
32	SyOpL1730	24.68
33	wPt-2757	25.23
34	cfd79	26.11
35	wPt-800213	26.52
36	wPt-7212	28.67
37	BS00011966	29.08
38	SyOpL2273	30.68
39	SyOpL2396	33.48
40	wmc307	34.77
41	wmc231	34.77
42	SyOpL305	36.98
43	SyOpL1361	39.18
44	wPt-8238	44.04
45	BS00070826	44.04
46	BS00022048	44.04
47	cfd28	46.11
48	SyOpL2303	46.98
49	BS00009513	47.43
50	wPt-6802	47.5
51	BS00010060	50.18
52	SyOpL2675	50.38
53	SyOpL1057	50.98
54	wPt-371	52.55
55	gwm285	54.06
56	BS00010405	55.3
57	wmc43	60.11
58	SNPPMOp6664/59	60.11
59	BS00023074	60.47
60	BS00003864	60.47
61	wPt-7486	60.59
62	wPt-9510	60.59
63	wmc78	60.59
64	barc1090	60.59
65	barc290	60.59
66	wPt-6239	60.59
67	wPt-6973	61.63
68	wPt-1159	61.63
69	wPt-4209	61.97
70	SNPPMOp1295258/65	62.41
71	SNPPMOp1750525/83	62.41
72	SNPPMOp400706/77	62.41
73	PAPMOp96539	62.41
74	gwm566	62.64
75	wPt-5105	63.96
76	BS00060014	63.96
77	wPt-7015	63.96
78	wPt-9170	63.96
79	wPt-6945	63.96
80	gpw7452	65.36
81	SyOpL1845	65.71
82	wPt-9432	65.81
83	SyOpL1930	66.01
84	barc1111	67.68
85	wmc777	68.07
86	BS00110445	68.2
87	SyOpL759	69.31
88	BS00015099	69.35
89	wmc625	69.39
90	barc1113	70.44
91	barc1044	70.44
92	wPt-6047	70.63
93	Sr12	70.63
94	wPt-5580	70.63
95	wPt-5946	70.63
96	SyOpL858	71.11
97	barc73	71.11
98	wPt-5390	71.11
99	gpw1120	71.11
100	wPt-0751	71.28
101	BS00094271	71.7
102	BS00010136	72.32
103	wPt-9433	73.23
104	wPt-5716	73.31
105	BS00078214	73.57
106	BS00009701	74.17
107	wPt-7229	74.3
108	BS00023030	74.72
109	wPt-2220	74.73
110	BS00000978	75.29
111	wPt-0544	75.47
112	wPt-0446	75.47
113	SNPPMOp1095216/14	76.71
114	PAPMM6398764	76.71
115	wPt-3327	76.71
116	wPt-1940	76.71
117	wmc418	80.11
118	SyOpL2017	80.11
119	wmc471	80.11
120	wPt-7502	80.11
121	wPt-10003	80.11
122	wPt-4082	80.11
123	BS00059417	81.61
124	BS00022403	83.8
125	BS00022245	84.75
126	SyOpL1217	85.11
127	BS00105878	88.15
128	barc334	91.23
129	wPt-1171	92.23
130	SyOpL2617	92.51
131	BS00097374	92.98
132	wPt-9912	93.42
133	wPt-8056	93.53
134	wPt-5769	93.78
135	wmc291	97.2
136	gwm108	97.53
137	wPt-5906	108.55
138	wPt-8096	108.55
139	wPt-3107	110.2
140	wPt-1804	112.53
141	SyOpL2491	115.21
142	SNPPMOp1526933/1	115.45
143	BS00070210	116.71
144	wPt-2439	120.82
145	barc77	121.35
146	SyOpL2194	121.35
147	gpw7258	126.29
148	SyOpL645	132.95
149	BS00021967	141.58
150	wPt-4412	143.18
151	gwm340	143.18
152	wPt-1311	143.53
153	wPt-4370	143.53
154	wPt-0365	144.22
155	gpw7220	144.22
156	wPt-0995	144.56
157	wPt-8352	144.56
158	gwm547	182.16

Chromosome 3D

1	BS00021930	0
2	wmc11	0
3	SNPPMOp561314/18	0
4	wPt-3921	0
5	BS00000447	5.95
6	gdm38	13.29
7	wmc11a	13.29
8	gdm99b	13.29
9	barc284	14.94
10	wPt-6909	14.94
11	wPt-5313	14.94
12	BS00010258	15.62
13	wmc43	16
14	wPt-9401	16.31
15	wPt-1741	16.31
16	gdm136a	16.44
17	wPt-3815	16.66
18	wPt-1336	16.66
19	wPt-6358	16.99
20	wPt-4476	18.98
21	wPt-2013	18.98
22	wPt-8463	19.34
23	w117472	28.74
24	gwm341	34.4
25	cfd62	38.84
26	BS00098058	38.91
27	wmc522	41.03
28	barc323	47.47
29	barc1161	51.87
30	gwm314	55.5
31	cfd120	57.83
32	BS00037276	59.19
33	wPt-6262	60.66
34	cfd9	62.13
35	wmc656	65
36	gwm383	65
37	cdf9	65
38	BS00021919	68.71
39	gdm99a	70.8
40	BS00098495	71.14
41	wPt-1440	76.2
42	wmc552	83.5
43	SNPPMOp1108885/33	83.5
44	gdm99c	85.98
45	BS00097485	86.43
46	wPt-10668	104.8
47	barc71	116.5
48	wPt-9525	139.45

Chromosome 4A

1	BS00108852	0
2	barc339	0
3	wmc707	0
4	barc1158	0.65
5	barc206	14.05
6	wPt-7524	16.46
7	gpw5133	17.61
8	gpw2271	18.3
9	gpw7196	18.3
10	gpw4315.2	20.29
11	wmc48	21.97
12	wmc420	21.97
13	wmc89a	22.34
14	BS00011378	22.61
15	barc1052	25.77
16	barc282	25.77
17	barc106	25.77
18	wPt-2788	28.55
19	SNPPMOp1043912/75	28.55
20	wPt-9057	28.95
21	wPt-5694	29.15
22	wPt-0117	29.15
23	wPt-7939	29.84
24	wPt-0817	30.16
25	wPt-7924	30.48
26	BS00022213	31.81
27	wmc650	35.05
28	wmc262	41.21
29	wPt-7001	41.3
30	wmc232	46.38
31	wPt-8078	48.09
32	wmc161_1	53.75
33	wmc161	56.05
34	BS00011939	56.05
35	BS00022837	56.05
36	BS00018651	56.05
37	BS00023220	56.05
38	wPt-1091	58.56
39	SNPPMOp10698/14	60.65
40	PAPMM6100799	60.65
41	BS00021957	64.58
42	wPt-6997	67.01
43	wPt-5857	67.01
44	wPt-9196	68.4
45	wPt-1743	68.73
46	wPt-5124	69.01
47	wmc283	69.05
48	SyOpL2079	69.91
49	barc78	70.68
50	wmc313	70.68
51	barc327	72.65
52	gwm350	73.29
53	BS00039766	73.39
54	BS00106888	73.39
55	gpw3094	73.94
56	gpw4334.1	74.29
57	wPt-5749	76.08
58	wPt-3349	83.68
59	SyOpL2131	83.98
60	wPt-3795	86.76
61	wPt-7807	86.76
62	wPt-2084	86.76
63	SNPPMOp37313/61	86.76
64	wPt-1362	86.76
65	wPt-7821	86.76
66	wPt-2319	86.76
67	wPt-9884	86.76
68	wPt-5212	86.76
69	wPt-1961	87.74
70	wPt-5172	87.74
71	wPt-0610	87.74
72	wPt-2951	87.74
73	wPt-4680	87.74
74	wPt-0150	87.74
75	wPt-3491	87.74
76	gwm160	89.05
77	SyOpL311	89.05
78	SNPPMOp1112908/84	89.05
79	SNPPMOp241208/61	89.05
80	SyOpL544	89.05
81	wPt-5434	101.28
82	wPt-4487	103.2
83	wPt-6390	103.2
84	wmc219	103.2
85	wPt-3402	113.43

Chromosome 4B

1	BS00022846	0
2	wPt-5559	0
3	wPt-8756	0
4	gwm495	0
5	SyOpL2051	0.54
6	wPt-9192	0.65
7	wPt-1046	3.3
8	wPt-8650	5.99
9	SyOpL1859	9.44
10	BS00106142	13.07
11	barc1142	13.57
12	SyOpL1441	22.24
13	barc1133	22.26
14	barc1179	23.66
15	barc1045	25.66
16	BS00010115	30.67
17	wPt-3015	30.97
18	BS00010830	36.14
19	cfd22	40.04
20	wPt-6149	42.62
21	BS00076259	44.52
22	wmc710	49.11
23	SyOpL189	49.11
24	wPt-1708	49.11
25	wPt-3991	50.78
26	wPt-5334	51.44
27	SNPPMM6863970/69	52.04
28	wmc511	52.04
29	tPt-0602	52.04
30	gwm6	52.04
31	gwm113	52.04
32	tPt-602	52.04
33	gwm513	57.67
34	BS00009774	58.97
35	SNPPMOp1023737/43	63.74
36	wmc125	63.74
37	wPt-5303	63.74
38	SyOpL1920	67.14
39	BS00014075	79.6
40	BS00073758	88.67
41	BS00083036	94.55
42	BS00022793	102.52
43	wPt-1115	102.93
44	BS00010015	108.25

Chromosome 4D

1	wmc285	0
2	wPt-5184	0
3	rht-4D	0
4	SNPPMOp157615/40	1.1
5	wPt-0648	3.2
6	wPt-4572	3.5
7	wPt-648	3.5
8	wPt-0941	16.85
9	gpw4315.1	17.88
10	wmc89b	19.24
11	wmc720	19.56
12	wPt-3178	22.95
13	wPt-2379	22.95
14	barc98	23.29
15	cfd71	23.29
16	gpw4087	23.63
17	gpw7414	23.95
18	gwm608	33.9
19	gpw342	34.17
20	wmc473	35.9
21	wmc331	35.94
22	wPt-5809	38.74
23	wPt-3058	38.74
24	BS00022113	39.41
25	gwm165	40.9
26	BS00023943	42.08
27	SNPPMOp1138764/28	45.2
28	wPt-7878	47.35
29	SNPPMOp1293165/14	51.7
30	gpw311	54.66
31	BS00023934	69.71
32	cfd84	69.9
33	SNPPMOp1286622/25	74.3
34	BS00089403	79.51
35	wPt-1412	81.29
36	gwm194	83.43

Chromosome 5A

1	gwm156A	0
2	gwm186	0
3	cfd39	0
4	barc141	1.29
5	BS00022110	2.65
6	BS00022500	3.28
7	BS00011278	3.86
8	wPt-10161	4.39
9	wmc713	4.39
10	wPt-9748	4.39
11	BS00089357	6.94
12	BS00011116	7.51
13	BS00087453	8.65
14	BS00051181	9.19
15	BS00011537	9.73
16	gwm293b	10.31
17	gpw2136	10.79
18	cos5F	11.59
19	gwm639	13.25
20	wPt-3563	15.13
21	gpw2172	15.96
22	wPt-3334	19.95
23	wPt-1903	19.95
24	wPt-5231	20.97
25	wmc327	27.9
26	wmc524	29.24
27	SyOpL2731	33.77
28	gwm291	38.81
29	gpw7007	39.4
30	gwm304	40.39
31	wmc705	40.39
32	wmc805	40.39
33	wPt-9887	40.39
34	SNPPMOp1978/35	40.47
35	PAPMOp105221	40.47
36	B1-5A	41.26
37	SNPPMOp717499/31	41.27
38	barc117	41.27
39	PAPMOp576041	41.87
40	wPt-4131	41.97
41	BS00021708	41.97
42	SNPPMOp314722/69	41.97
43	wPt-4249	41.97
44	PAPMOp1059158	41.97
45	wPt-2768	41.97
46	wPt-6048	41.97
47	SyOpL643	42.27
48	wPt-6990	44.04
49	gmw304	45.27
50	gpw2273	50.37
51	wPt-8794	55.01
52	SNPPMOp1096021/17	61.79
53	gwm617	62.27
54	wmc415	65.27
55	BS00029413	76.07
56	SNPPMOp115775/59	110.29
57	wPt-5096	126.59
58	wmc727	126.59
59	SyOpL778	126.59
60	wPt-733461	126.63
61	wPt-5142	144.88
62	BS00082218	145.37
63	BS00091080	172.16
64	BS00010234	196.52

Chromosome 5B

1	BS00009264	0
2	wPt-1302	19.81
3	SyOpL283	20.51
4	SyOpL250	20.51
5	gwm234	22.54
6	BS00102828	22.54
7	BS00015136	22.54
8	wPt-8406	27.44
9	wPt-3873	29.16
10	wPt-1149	30.26
11	wPt-5346	33.78
12	wPt-1589	36.74
13	SNPPMOp185131/43	37.41
14	BS00062617	37.67
15	wPt-2586	39.55
16	BS00018917	40.72
17	SNPPMOp201244/17	42.91
18	wPt-4996	42.91
19	barc4	42.91
20	SNPPMOp130896/16	44.71
21	gwm544	45.54
22	BS00107222	48.42
23	BS00035899	54.21
24	gwm213	54.88
25	gwm371	57.54
26	SNPPMOp445009/5	59.61
27	barc74	63.95
28	BS00003876	64.53
29	wmc415	68.54
30	PAPMM6100537	70.73
31	wPt-2607	72.75
32	BS00091302	76.36
33	BS00011386	76.36
34	wPt-1250	76.36
35	wPt-3457	77.93
36	SNPPMOp1472/52	77.93
37	wPt-9356	79.62
38	BS00010952	82.65
39	SyOpL2437	84.24
40	wPt-3661	85.43
41	SNPPMOp332417/44	90.64
42	BS00009414	95.11
43	SNPPMOp468860/33	95.34
44	wPt-1733	96.44
45	wmc289	96.54
46	SyOpL1005	96.54
47	wPt-5896	96.54
48	wPt-6014	99.79
49	wPt-3030	103.11
50	BS00000592	104.22
51	wPt-0750	104.57
52	gdm136b	109.74
53	barc275	110.1
54	gwm408	111.68
55	BS00022963	112.25
56	gwm604	117.07
57	SNPPMOp1640210/20	118.14
58	SyOpL1960	118.14
59	SNPPMOp558187/29	118.14
60	barc142	118.54
61	rPt-3114	119.44
62	wPt-9598	119.64
63	wPt-1482	119.64
64	wPt-9103	119.64
65	wPt-0935	119.98
66	wPt-9205	119.98
67	wPt-8094	119.98
68	wmc99	123.95
69	wmc235	127.36
70	barc232	128.09
71	barc266	129.8
72	gwm142	135.52
73	barc59	138.54
74	BS00003655	140.64
75	BS00021960	144.27
76	wPt-4418	149.6
77	BS00021709	150.58
78	wPt-0927	153.56
79	barc243	155.57
80	barc156	158.68
81	wPt-9815	168.66
82	gwm118	174.39
83	wPt-3922	175.79
84	wPt-9116	176.13
85	wPt-7665	176.13
86	wPt-2373	176.13
87	barc1120	176.56
88	wPt-0484	177.42
89	wPt-0837	193.09
90	wPt-9894	193.09
91	wPt-4898	193.09
92	wPt-9027	193.09

Chromosome 5D

1	wPt-5032	0
2	cfd18	0
3	Pina	8.02
4	Pinb	9.36
5	wPt-6225	24.2
6	gwm358	29.01
7	BS00022100	29.01
8	BS00021901	29.59
9	gwm159	29.59
10	BS00041838	30.21
11	BS00022067	33.64
12	gwm174	56.2
13	gwm182	60.2
14	SrHD	85.3
15	gwm212	106.53
16	BS00022228	121.33

Chromosome 6A

1	gwm617_2	0
2	SNPPMOp1519638/55	0
3	wmc417_1	8.6
4	wmc570	26.2
5	BS00011125	45.04
6	PAPMM6137386	51.2
7	wPt-1664	51.88
8	wPt-9382	51.88
9	wPt-0832	54.35
10	wPt-7475	54.68
11	wPt-1377	54.68
12	BS00083630	55.92
13	wPt-669498	60.35
14	gwm334	65
15	BS00010933	65
16	wPt-7623	65
17	wPt-6520	65
18	SNPPMOp10333/21	65
19	wPt-4016	65
20	wPt-8266	78.21
21	wPt-2573	85.96
22	wPt7127	85.96
23	wPt-9690	85.96
24	gpw3087	87.69
25	wPt-0959	88.72
26	wPt-7599	89.08
27	wPt-0357	89.43
28	wPt-8833	89.43
29	wPt-3091	89.43
30	wmc807	104
31	wPt-7373	108.93
32	barc144	112.91
33	BS00082104	119.3
34	gwm570	119.3
35	wPt-5601	125.33
36	wPt-8662	133.08
37	wPt-5310	134.82
38	BS00021965	140.9
39	wmc417	149
40	gwm617	158
41	gwm427	162.76
42	wPt-6696	164.93
43	BS00003339	167
44	wPt-4229	167.28
45	wPt-9474	167.28
46	wPt-1661	167.63
47	wPt-1642	167.63
48	wPt-9976	167.63
49	wPt-4445	167.63
50	gpw7433.2	169.03
51	wPt-7204	169.37
52	wPt-1695	169.37
53	wPt-8124	169.37
54	wPt-6268	173.84
55	wmc254	211
56	wmc59	219

Chromosome 6B

1	wPt-2175	0
2	wPt-7745	0
3	wPt-8721	0
4	barc1136	0
5	wmc397	0
6	gpw1079	0
7	wPt-3774	0.98
8	wPt-7662	0.98
9	BS00003785	1.39
10	BS00003792	1.39
11	BS00009795	1.39
12	gwm88	1.47
13	wPt-3376	1.87
14	wPt-4930	1.87
15	barc1159	2.17
16	barc1093	2.5
17	gwm58	2.83
18	gpw5076	4.15
19	barc354	4.15
20	wPt-9532	5.2
21	wPt-7777	6.18
22	wPt-3581	6.18
23	wPt-6994	6.6
24	wPt-4720	6.6
25	wPt-4283	6.6
26	wPt-7150	6.6
27	wPt-5234	6.6
28	wPt-2689	6.6
29	wPt-0245	6.6
30	wPt-1852	6.61
31	wPt-3304	6.61
32	wPt-7207	6.94
33	wPt-8239	6.95
34	wPt-4924	7.18
35	wPt-1922	7.27
36	wPt-3130	7.27
37	wPt-9990	7.27
38	wPt-4386	7.27
39	wPt-8015	7.27
40	wPt-3060	8.88
41	wPt-4648	9.21
42	BS00010443	11.14
43	gwm174	13.41
44	wPt-5596	29.9
45	BS00010415	32.29
46	cfd13	32.29
47	wPt-5333	32.29
48	BS00003852	33.01
49	BS00022668	44.48
50	wPt-3733	44.48
51	gwm518	44.48
52	barc1169	44.48
53	wPt-2964	44.48
54	wPt-7636	44.48
55	BS00086964	50.86
56	barc14a	52.76
57	wPt-5461	53.18
58	wPt-4164	56.5
59	wPt-9952	56.5
60	wPt-6116	56.5
61	wPt-3045	57.15
62	wPt-1264	57.15
63	wPt-8268	57.51
64	BS00010993	57.68
65	wPt-5037	57.98
66	wmc152	58.67
67	BS00109036	58.87
68	BS00023187	60.57
69	wPt-6023	61.08
70	wPt-6802	64.35
71	gwm200a	64.97
72	wmc417	78.58
73	BS00022480	79.5
74	wPt-5480	101.85
75	BS00011458	102.63
76	BS00085589	102.63
77	wPt-0171	106.82
78	wPt-1541	107.11
79	BS00010618	107.41
80	wPt-9256	110.09
81	BS00023066	110.4
82	BS00110651	126.18
83	wPt-732061	128.07

Chromosome 6D

1	BS00003785	0
2	BS00003792	0
3	BS00009795	0
4	wPt-6994	0
5	SNPPMM61159750/20	0
6	wPt-3774	0
7	PAPMM6106536	0
8	wPt-7777	0
9	wPt-7662	0
10	wPt-1922	0
11	wPt-9532	0
12	wPt-4720	0
13	wPt-4283	0
14	wPt-7207	0
15	wPt-3130	0.01
16	wPt-1852	0.01
17	wPt-9990	0.01
18	wPt-8239	0.01
19	wPt-3304	0.01
20	SyOpL281	1
21	SyOpL2232	3.4
22	SyOpL362	4.6
23	BS00010443	5.45
24	SyOpL1457	7.3
25	SyOpL1451	10
26	SyOpL1919	10
27	wPt-3424	11.3
28	SNPPMOp1239709/60	11.3
29	SNPPMOp157697/36	17.8
30	wPt-2175	18.2
31	SyOpL1247	18.5
32	SyOpL14	19.8
33	wPt-5596	22.95
34	BS00010415	26.6
35	cfd13	26.6
36	SNPPMOp1227776/50	26.6
37	wPt-5333	27.62
38	BS00003852	30.05
39	gwm518	40.6
40	SNPPMOp103652/20	40.6
41	SyOpL1051	41.2
42	BS00022668	41.52
43	wPt-3733	41.52
44	BS00086964	47.9
45	wPt-5461	48.31
46	SNPPMOp261873/83	51.4
47	wPt-5037	53.11
48	BS00010993	54.72
49	BS00109036	55.91
50	BS00023187	57.61
51	wmc152	62.6
52	SNPPMOp3332865/80	62.6
53	wmc417-2	62.6
54	SNPPMOp372691/21	62.6
55	wPt-6802	63.37
56	SyOpL1225	73.4
57	SyOpL740	74.4
58	SyOpL313	75
59	BS00022480	76.54
60	PAPMOp86183	77.4
61	wmc417	77.6
62	BS00011458	99.67
63	BS00085589	99.67
64	wPt-5480	100.87
65	BS00010618	104.45
66	wPt-0171	105.84
67	wPt-1541	106.13
68	BS00023066	107.44
69	wPt-9256	109.11
70	BS00110651	123.22
71	wPt-732061	127.09

Chromosome 7A

1	wPt-9651	0
2	wPt-0433	0
3	cfa2174	0
4	wmc479	7.83
5	gwm635a	10.55
6	BS00022406	10.55
7	BS00022082	10.55
8	wmc83	11.68
9	wPt-672171	13.45
10	wPt-8043	13.55
11	gwm332	13.6
12	wPt-2371	13.95
13	barc1004	14.13
14	barc70	14.55
15	barc22	14.79
16	wPt-9207	15.24
17	gpw3127	15.36
18	wPt-7448	17.26
19	wPt-1639	17.26
20	wPt-6668	20.06
21	SNPPMOp1069993/13	20.17
22	wPt-4748	20.17
23	wPt742244	20.17
24	SNPPMOp410647/16	21.77
25	SyOpL1199	22.47
26	wPt-6013	23.32
27	wPt-5558	23.32
28	wPt-4553	23.69
29	wPt-0961	23.69
30	BS00010988	24.29
31	BS00013872	29.85
32	wPt-5257	31.31
33	barc154	31.51
34	barc127	33.88
35	wmc283.1	34.61
36	wPt-9796	35.69
37	wPt-3883	35.69
38	wPt-7186	36.28
39	BS00002556	36.33
40	wPt8418	39.01
41	gwm60	41.11
42	BS00111112	41.83
43	wmc283_1	44.17
44	wmc283	44.37
45	gwm471	46.39
46	wPt-744715	49.75
47	SNPPMOp2675910/36	52.37
48	cfa2028	52.37
49	wPt-4515	52.37
50	SyOpL2262	53.87
51	BS00021771	63.13
52	barc121_1	75.57
53	BS00000847	80.37
54	barc121	80.37
55	wmc607	81.37
56	SyOpL2323	94.27
57	BS00078174	96.89
58	SyOpL2300	98.57
59	SyOpL896	109.47
60	cfa2019	111.37
61	wmc633	112.37
62	cfa2040_3	112.57
63	SyOpL2086	121.67
64	cfa2040a	123.37
65	wPt-8670	123.37
66	SyOpL1907	124.07
67	SyOpL1876	126.47
68	BS00010809	141.14
69	wPt-741686	151.37
70	wPt-6495	155.27
71	BS00022560	157.71
72	wPt-7763	168.97
73	BS00073988	171.13
74	wPt-8846	174.15
75	BS00011622	178.56
76	BS00081108	192.28
77	BS00023200	203.33

Chromosome 7B

1	wmc323	0
2	barc279	16.09
3	wPt-7318	29.59
4	gwm537	40
5	gwm573	53
6	wPt-7653	53
7	gpw3188	53
8	gpw4444	53
9	gpw7342	53
10	gwm46	53
11	wPt-4863	53
12	wPt-0963	53
13	wPt-4230	53
14	wmc335	53
15	gwm297	53
16	barc289	53
17	barc276b	56.59
18	barc276c	57.69
19	wPt-7934	60.51
20	wPt-6498	60.51
21	wPt-1149	66.54
22	wPt-2572	67.65
23	wPt-3873	69.6
24	wPt-6372	69.6
25	wPt-2305	72.19
26	wPt0312	72.19
27	barc1014	73.22
28	barc278	75.96
29	wPt-3730	77.73
30	wPt-4025	77.73
31	BS00011069	79.15
32	wPt-2994	80.59
33	wPt-2273	80.59
34	BS00010500	81.39
35	BS00022106	82.63
36	wPt-7351	85.31
37	BS00065735	86.11
38	wPt-1826	91.23
39	wmc517	92
40	wPt-3723	92
41	wPt-9925	93.91
42	SNPPMM6777963/4	94.54
43	gpw8040	98.7
44	wPt-8007	99.34
45	wPt-0600	101.54
46	SyOpL143	101.54
47	wPt-3190	101.54
48	wPt-4258	103
49	SyOpL1352	105.14
50	BS00023023	106.8
51	SNPPMOp402574/42	108.24
52	wPt-5343	108.54
53	BS00010251	108.54
54	SNPPMOp108828/50	108.54
55	SyOpL1351	108.54
56	wPt-0194	114.82
57	BS00074359	115.78
58	wPt-2356	118.97
59	wPt-4300	119.31
60	BS00009464	122.29
61	BS00011065	123.66
62	wPt-5462	133.74
63	gwm577	136
64	gwm111	141.19
65	BS00010142	153.26
66	wPt-9488	154.4

Chromosome 7D

1	cfa2040	0
2	wPt-2565	0
3	wPt-665260	3.72
4	gwm635	8.32
5	barc121_2	11.82
6	gwm130	17.21
7	gwm437	17.21
8	gpw5199	17.21
9	wPt-8798	21.1
10	gpw1142	23.39
11	barc128b	25.86
12	gwm473	30.31
13	SNPPMOp1043882/16	31.49
14	cfd21_1	32.92
15	Sr57	33.2
16	w117337	37.29
17	BS00022463	41.42
18	wPt-1100	45.14
19	wPt-3328	45.14
20	barc1104	45.21
21	barc352	46.8
22	wPt-7171	47.42
23	SNPPMOp101458/70	47.42
24	wPt-789	47.42
25	csLV34	48.02
26	SNPPMOp1034549/16	48.02
27	cssfr5	49.32
28	wmc463	49.32
29	gwm428	56.48
30	cfd21	57.32
31	wPt-9822	61.29
32	wPt-2258	61.29
33	SNPPMM62481791/38	61.29
34	SyOpL1368	61.29
35	wPt-7763	61.29
36	wPt-731810	61.29
37	wPt-7642	61.29
38	cfd66	63.19
39	gdm145	64.97
40	wmc157	69.07
41	BS00021987	69.16
42	BS00022511	69.16
43	BS00022721	69.16
44	BS00022875	69.16
45	BS00023045	69.16
46	BS00003945	69.74
47	BS00010071	69.74
48	BS00012122	69.74
49	BS00021745	69.74
50	BS00023184	69.74
51	BS00024032	69.74
52	BS00024103	69.74
53	BS00009623	70.3
54	BS00011639	70.3
55	BS00079734	70.3
56	wPt-2054	70.32
57	barc97	72.01
58	cfd41	78.54
59	barc121	84.32
60	wPt-1859	84.32
61	gpw5192	84.32
62	wPt-6716	84.32
63	gpw7683	103.13
64	wPt-3923	115.56
65	wPt-5674	115.56
66	wPt-664017	116.52
67	gwm635_1	117.9
68	gwm37	120.32
69	cfa2040b	122.32
